# Histopathological Spectrum of the Human Appendix with Aging and Adiposity: A Forensic Autopsy Study

**DOI:** 10.3390/diagnostics16213495

**Published:** 2026-10-25

**Authors:** Ahmet Uğur Akman, Abdülkadir Kutlu, Ahmet Depreli, Deniz Demirkıran, Kamil Hakan Doğan

**Affiliations:** 1 Department of Histology and Embryology, Faculty of Medicine, Amasya University, 05100 Amasya, Türkiye; abdulkadir.kutlu@amasya.edu.tr; 2 Department of Forensic Medicine, Faculty of Medicine, Selçuk University, 42130 Konya, Türkiye; ahmetdep@gmail.com (A.D.); drhakan2000@gmail.com (K.H.D.); 3 Department of Pathology, Beyhekim Training and Research Hospital, Health Sciences University, 42130 Konya, Türkiye; denizkarasoy@hotmail.com

**Keywords:** appendix, forensic autopsy, medico-legal evaluation, aging, adiposity, fibrosis, histopathology, submucosal adipose accumulation

## Abstract

**Background:** Histological variation in the vermiform appendix in forensic autopsy populations remains incompletely characterized. This study evaluated age- and adiposity-related histopathological patterns and their potential medico-legal relevance. **Methods:** This prospective cross-sectional study included 101 consecutive adult forensic autopsy cases. Appendiceal specimens were assessed semi-quantitatively for lymphoid nodules, fibrosis, luminal narrowing, and submucosal adipose accumulation, with morphometric assessment of the submucosa-to-muscularis propria ratio. Associations with age and adiposity measures were examined using correlation analyses and multivariable ordinal regression. Postmortem interval (PMI) and natural versus non-natural cause of death were evaluated in sensitivity analyses. **Results:** Advancing age was associated with lower lymphoid nodule scores (ρ = −0.301, *p* = 0.002) and higher fibrosis (ρ = 0.403, *p* < 0.001), luminal-status (ρ = 0.228, *p* = 0.022), and submucosal-adipose-accumulation scores (ρ = 0.302, *p* = 0.002). In adjusted models, age remained associated with lower lymphoid scores (OR = 0.971, 95% CI 0.951–0.992) and higher submucosal adipose accumulation (OR = 1.027, 95% CI 1.007–1.048), while fibrosis showed threshold-specific age associations (ORs = 1.039 and 1.087; 95% CIs 1.013–1.065 and 1.050–1.125). Unadjusted associations between adiposity measures and histopathological outcomes were modest. Greater abdominal subcutaneous fat thickness was associated with higher luminal-status (OR = 1.212, 95% CI 1.027–1.431) and submucosal-adipose-accumulation scores (OR = 1.203, 95% CI 1.022–1.416) after adjustment for age and sex. In the three-level luminal sensitivity model, the age estimate was essentially unchanged after adding PMI, and PMI was not significant. After additional adjustment for cause of death, age associations with fibrosis and submucosal adipose accumulation remained significant, whereas those with lymphoid nodule score and luminal status did not. **Conclusions:** Age showed the most consistent associations with appendiceal histological variation, particularly fibrosis and submucosal adipose accumulation. Adiposity-related associations were modest and measure-dependent. These findings provide descriptive forensic context but do not establish diagnostic thresholds or causality.

## 1. Introduction

Although the vermiform appendix is only infrequently the focus of forensic autopsy, it may be encountered during routine abdominal examination; however, histological sampling of the appendix is not necessarily a routine component of all medico-legal autopsies. Published autopsy practice data indicate that histological examination of the appendix may be selective rather than routine, particularly when gross abnormalities are identified [1]. Appendiceal findings seldom constitute the primary investigative target in forensic practice; however, when the appendix is evaluated as part of a comprehensive medico-legal workup—especially in deaths with unexplained abdominal findings or when appendiceal disease must be ruled out as a contributing factor—histopathological examination may provide additional diagnostic information within the broader autopsy context [2].

Forensic autopsy material differs fundamentally from surgical pathology specimens, which are obtained on the basis of a clinical indication for appendectomy. This distinction provides an opportunity to characterize appendiceal histological variation in specimens obtained independently of a clinical indication for appendectomy. Such an advantage is especially pertinent for forensic pathologists, who must interpret appendiceal findings in the absence of a clinical history pointing to a specific gastrointestinal diagnosis and determine their relevance within the broader medico-legal examination.

The appendix contains a prominent lymphoid compartment that undergoes age-related involution. Human histological studies have demonstrated progressive reductions in appendiceal lymphoid tissue with advancing age [3,4], while a recent small study reported more frequent lymphoid follicle reduction in aged appendiceal samples [5]. Beyond its lymphoid component, the appendix has recognized immunological and microbial functions, including roles in mucosal immunity and maintenance of the intestinal microbiota [6,7]. The distribution of immunoglobulin-producing cells differs between the appendix and the large intestine, suggesting regional specialization of mucosal immune responses [8]. Adipose tissue also undergoes age- and depot-dependent remodeling, and regional measures of abdominal adiposity may provide information that is not fully captured by generalized anthropometric measures [9,10]. These considerations provide a rationale for evaluating both systemic and regional adiposity measures in relation to microscopic adipose accumulation within the appendiceal wall.

Although recent large surgical series and contemporary pathology reviews have substantially expanded the characterization of appendiceal histopathological findings, including incidental neoplastic and non-neoplastic lesions [11,12,13], systematic studies evaluating age- and adiposity-associated histological variation specifically in forensic autopsy populations remain scarce. Contemporary reviews describe a broad non-neoplastic histopathological spectrum of the appendix, while recent surgical series have demonstrated that incidental findings, including fibrous obliteration, may be identified histologically in appendectomy specimens [11,12]. A recent 15-center Turkish study of 40,286 adult appendectomy specimens further illustrates the breadth of contemporary appendiceal histopathology but remains based on surgically selected material rather than forensic autopsy tissue [13]. Consequently, the background range of lymphoid, fibrous, luminal, and adipose variation across adulthood remains incompletely characterized in the forensic setting.

In the present investigation, based on a consecutive series of forensic autopsy cases at the Council of Forensic Medicine, Konya Group Chairmanship, Morgue Specialization Department, we performed a systematic evaluation of age- and adiposity-associated histopathological findings of the appendix—encompassing lymphoid nodules, fibrosis, luminal status, and submucosal adipose accumulation—alongside morphometric assessment of the submucosa-to-muscularis propria (SM/MP) ratio. By characterizing these findings in a forensic autopsy population sampled independently of clinical indication, we aimed to provide morphological context for interpreting age- and adiposity-associated appendiceal variation within the broader medico-legal examination, without proposing diagnostic thresholds or criteria for distinguishing physiological variation from disease.

## 2. Materials and Methods

### 2.1. Study Design and Study Population

This prospective cross-sectional observational autopsy study with consecutive standardized data and tissue collection was conducted using forensic autopsy cases examined at the Council of Forensic Medicine, Konya Group Chairmanship, Morgue Specialization Department between 1 January 2026 and 31 March 2026.

During the study period, 136 adult forensic autopsy cases were assessed for eligibility. Cases were excluded if they had congenital malformations of the appendix or adjacent gastrointestinal structures, advanced appendiceal putrefaction or autolysis precluding reliable macroscopic or histopathological evaluation, abdominal trauma involving the appendix or adjacent gastrointestinal structures that could compromise macroscopic or histological assessment, penetrating or sharp-force injury involving the abdominal region, documented diabetes mellitus based on the available medical history and medical records, a history of colorectal malignancy, an undetermined cause of death, or referral to a higher forensic medical authority that prevented completion of the standardized examination protocol. Macroscopic or histopathological evidence of acute appendicitis, appendiceal neoplasia, marked active inflammation, or inflammatory bowel disease involving the appendix constituted predefined exclusion criteria.

Of the 136 cases assessed, 35 were excluded: abdominal trauma (*n* = 8), penetrating or sharp-force abdominal injury (*n* = 6), advanced appendiceal putrefaction/autolysis precluding reliable histopathological evaluation (*n* = 4), a history of colorectal malignancy (*n* = 3), and documented diabetes mellitus (*n* = 14). The remaining 101 eligible cases constituted the final analytical cohort. Eligibility for the final analytical cohort was determined on the basis of the predefined clinical, macroscopic, and histopathological exclusion criteria. None of the assessed cases met the predefined exclusion criteria for congenital appendiceal malformations, acute appendicitis, appendiceal neoplasia, marked active inflammation, inflammatory bowel disease involving the appendix, an undetermined cause of death, or referral to a higher forensic medical authority.

The study size was determined by the number of consecutive eligible adult forensic autopsy cases available during the predefined study period; no formal a priori sample-size calculation was performed. The age range was 18–83 years, and the postmortem interval (PMI) ranged from 2 to 9 h among the 101 included cases. PMI was defined as the interval between the documented or estimated time of death recorded in the official medico-legal case file and the start of the autopsy. Depending on the circumstances of death, the time of death was based on available clinical, scene, or investigative documentation rather than necessarily representing a directly observed time of death. The case-selection process is summarized in Supplementary Figure S1.

The appendix was examined as part of the routine autopsy procedure. The entire appendix was removed and examined macroscopically. In all cases, the appendix was transected transversely at its midpoint, and a 5 mm thick full-thickness tissue specimen was obtained from the mid-appendiceal region for histopathological evaluation. Tissue sampling was performed only after completion of the standard macroscopic examination and did not alter the routine medico-legal investigation. The study population therefore consisted of appendices sampled independently of a clinical indication for appendectomy.

Cause-of-death information was available for all 101 cases. Causes of death were grouped into five principal categories—asphyxia, natural death, trauma, poisoning, and other external causes—while the specific cause within each category was retained for descriptive reporting.

Ethical approval for this observational autopsy study was obtained from the Education and Scientific Research Commission of the Council of Forensic Medicine, Ministry of Justice, Republic of Türkiye (Decision No: 21589509/2025/1455, Date: 11 November 2025) and the Local Ethics Committee of Selçuk University Faculty of Medicine (Decision No: 2025/704, Date: 2 December 2025). Histopathological and morphometric evaluations were performed on appendix specimens obtained from adult forensic autopsy cases. All specimens and anthropometric data were recorded systematically and consecutively following a standardized protocol. Both approvals were obtained before data collection. There were no missing data for the variables included in the present analyses, and all 101 cases contributed to the histopathological, morphometric, correlation, and regression analyses.

### 2.2. Anthropometric Measurements and Tissue Sampling

Anthropometric measurements were obtained during the routine autopsy examination. Body weight, stature, and waist circumference were measured before dissection. ASFT was measured after opening of the abdominal cavity, as described below. All bodies were received at the autopsy unit without clothing; garments had been removed at the scene or hospital before transfer to the unit.

Body weight was measured before autopsy using the weighing scale available in the autopsy unit. Stature was measured before the autopsy procedure with the body in the supine position using a standard measuring tape, from the vertex of the head to the heel. Body mass index (BMI) was calculated as body weight in kilograms divided by height in meters squared. No cases had clinically apparent generalized edema or major tissue loss that would be expected to materially affect body-weight or stature measurements. Waist circumference was measured at the midpoint between the lower costal margin and the iliac crest. Replicate measurements were not separately documented in the study records; therefore, repeat-measurement reliability could not be assessed.

**Figure 1:**
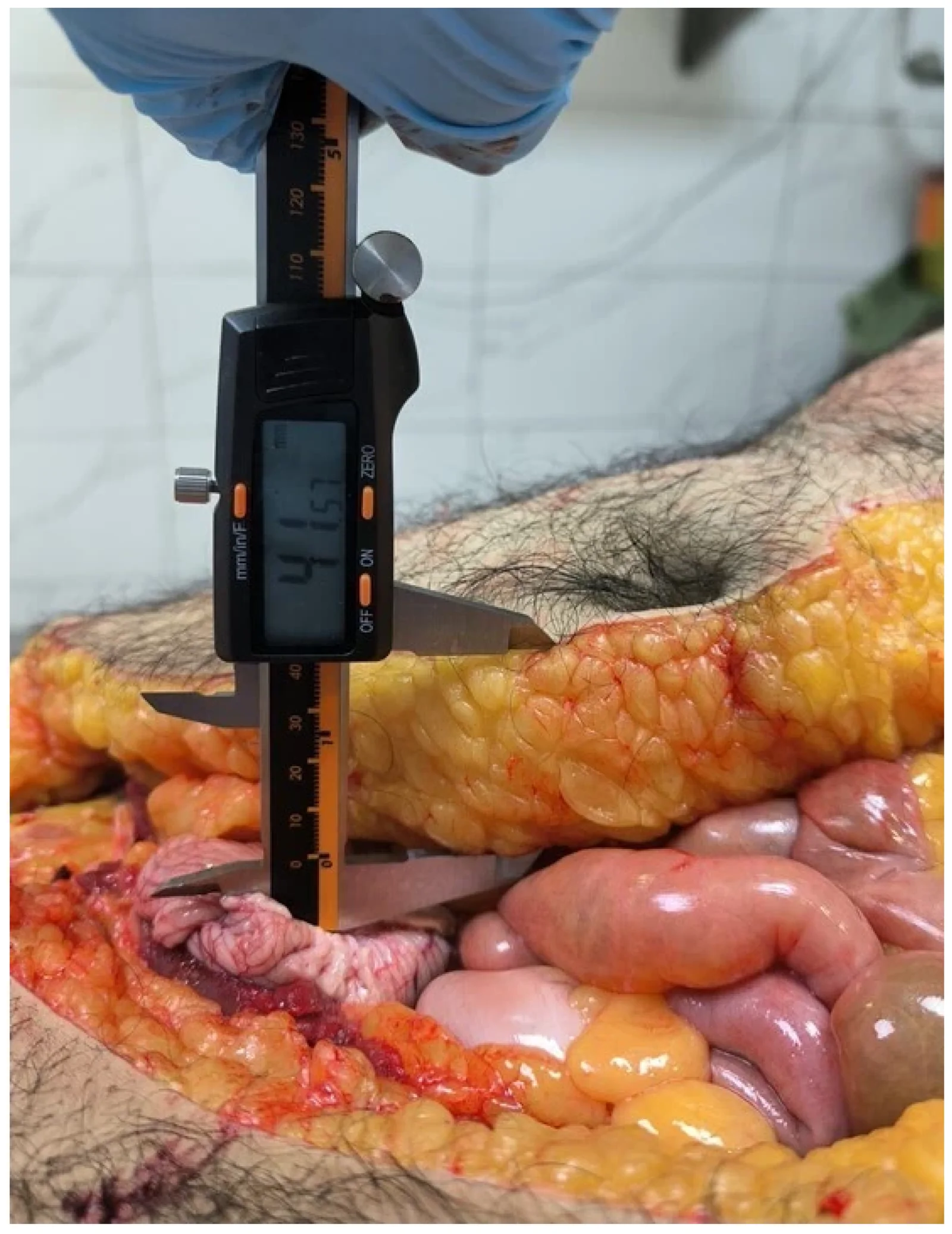
Measurement of abdominal subcutaneous fat thickness. Measurement of abdominal subcutaneous fat thickness (ASFT) using a digital caliper on the exposed anterior abdominal wall after opening of the abdominal cavity. The measurement was obtained at a standardized site 1 cm inferior to the umbilicus, between the superficial and macroscopically identifiable deep boundaries of the subcutaneous adipose tissue, while avoiding tissue compression.

Abdominal subcutaneous fat thickness (ASFT) was measured during autopsy on the exposed anterior abdominal wall after opening of the abdominal cavity. The measurement was performed using a digital caliper at the standardized location 1 cm inferior to the umbilicus. Subcutaneous adipose thickness was defined as the distance from the superficial boundary of the subcutaneous adipose tissue to the macroscopically identifiable deep boundary at which the adipose tissue ended. Care was taken to avoid compression of the adipose tissue during measurement. The digital caliper had a technical resolution of 0.01 mm; this value reflects the resolution of the instrument and should not be interpreted as the biological precision or reproducibility of the measurement. Figure 1 illustrates the measurement procedure.

During forensic autopsy, the abdominal cavity was opened and the abdominal organs were examined macroscopically as part of the standard autopsy procedure. Histological sampling of the appendix, however, is not a routine procedure in every forensic autopsy. In the present study, appendiceal sampling was performed systematically in all included cases as part of the predefined research protocol. The appendix was anatomically identified during abdominal examination, macroscopically assessed, and then sampled specifically for histopathological examination.

**Figure 2:**
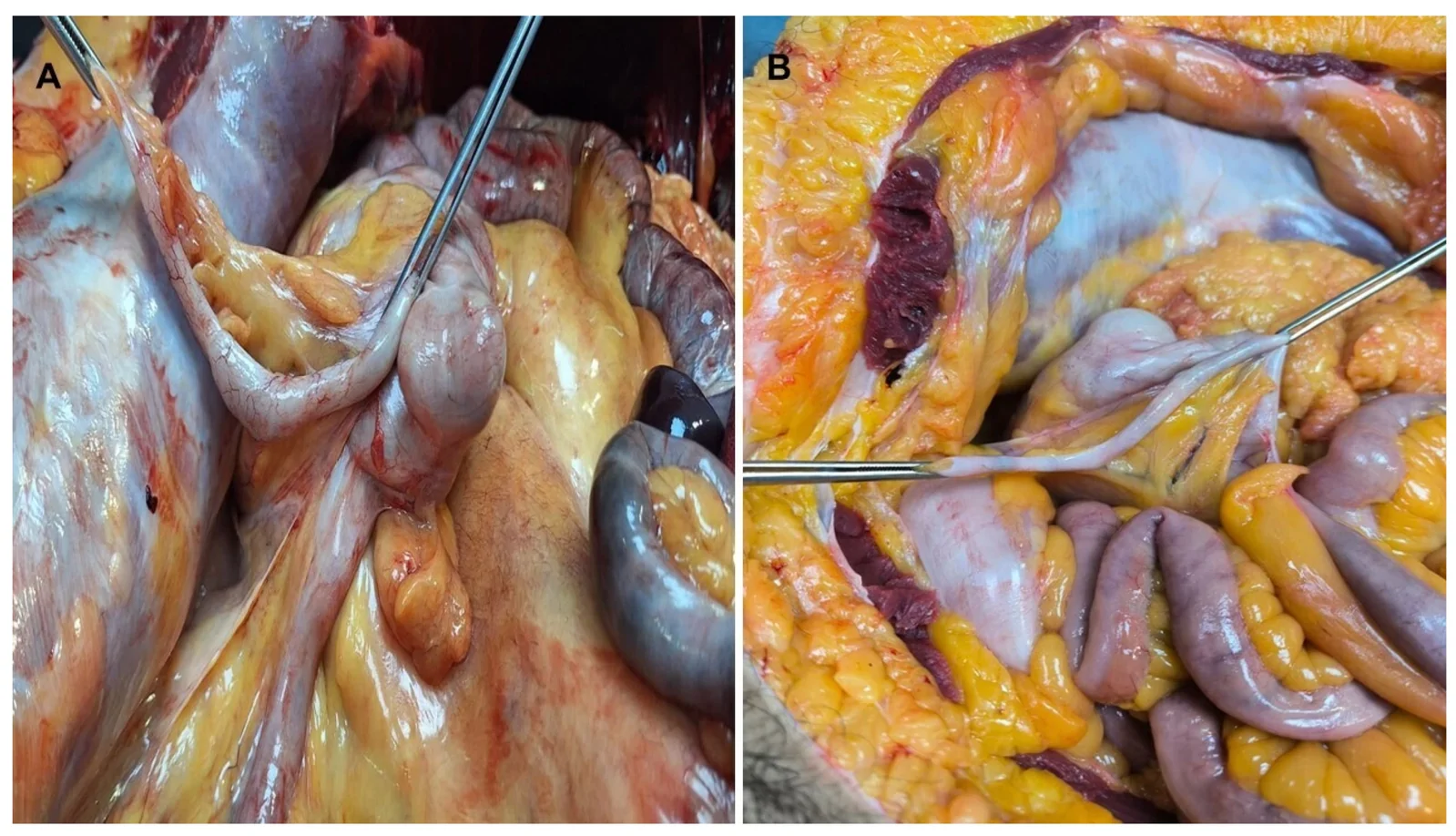
Anatomical identification and exposure of the appendix in two representative cases. (**A**) The appendix is identified at its origin from the cecum and gently elevated using forceps. (**B**) In a different case, the appendix is exposed and isolated, demonstrating its relationship with surrounding mesenteric adipose tissue and intestinal loops. The images illustrate the anatomical context in which the appendix was identified and sampled according to the study-specific standardized protocol.

A representative full-thickness tissue specimen was obtained from the midpoint of the appendix after transverse transection, following macroscopic examination (Figure 2). Sampling from the mid-appendiceal region was used to minimize potential variation associated with the appendiceal base and tip. Tissue specimens were subsequently transported to the Histology and Embryology Laboratory of Amasya University Faculty of Medicine for histopathological processing.

### 2.3. Histopathological Evaluation

The standardized mid-appendiceal tissue specimens obtained as described above were fixed in 10% neutral buffered formalin for 24 h, routinely processed, and embedded in paraffin. From each paraffin block, four serial 5-μm sections were prepared: three sections for hematoxylin and eosin (H&E) staining and one section for Masson’s trichrome staining. Although sampling was standardized to a full-thickness transverse specimen obtained from the midpoint of the appendix in all cases, total evaluable mucosal length and tissue area were not recorded as quantitative variables; therefore, lymphoid nodule counts were not normalized to an anatomical denominator. The three serial H&E sections were reviewed to ensure adequate tissue representation and evaluability; however, semi-quantitative histopathological scoring was performed on a single representative, well-oriented H&E section selected for each case. The same H&E section was used to assess lymphoid nodules, fibrosis, luminal status, and submucosal adipose accumulation. Masson’s trichrome staining was performed in all cases to facilitate the identification and visualization of collagen-rich areas and to support the morphological assessment of fibrosis.

Before application of the semi-quantitative scoring system, the H&E-stained sections were reviewed for evidence of specific appendiceal pathology. Acute appendicitis, appendiceal neoplasia, marked active inflammation, and histological involvement compatible with inflammatory bowel disease constituted predefined histopathological exclusion criteria. No case was excluded on the basis of these histopathological criteria. Histopathological scoring was therefore performed after confirming the absence of these specific pathological processes in the analyzed cohort.

Histopathological evaluation was performed using a predefined scoring protocol with a ZEISS Primostar 3 light microscope (Carl Zeiss Suzhou Co., Ltd., Suzhou, China). Representative photomicrographs were acquired using a 4× objective (total magnification, ×40) during the histopathological evaluation, before statistical analyses were performed. Histopathological scores were established prior to statistical analysis. Initial scoring was independently performed by two experienced histologists who were unaware of the demographic and anthropometric characteristics of the cases. Subsequently, all specimens were reviewed by an experienced pathologist who was blinded to the demographic and anthropometric data.

Four histopathological parameters were evaluated: lymphoid nodules, fibrosis, luminal status, and submucosal adipose accumulation. Each parameter was assessed using a predefined four-point ordinal scale ranging from 0 to 3. When discrepancies occurred between observers, the slides were jointly reviewed using a multi-headed microscope, and a consensus score was established.

Inter-observer agreement between the initial observers was assessed using Cohen’s kappa coefficient. Agreement was substantial to excellent for lymphoid nodules (κ = 0.81), fibrosis (κ = 0.79), luminal status (κ = 0.84), and submucosal adipose accumulation (κ = 0.76).

### 2.4. Scoring System

A study-specific semi-quantitative histopathological scoring system was used to assess structural changes in the appendiceal wall. Semi-quantitative histological grading approaches have been used in gastrointestinal pathology to standardize the assessment of morphological changes [14]. Four histopathological parameters—lymphoid nodules, fibrosis, luminal status, and submucosal adipose accumulation—were graded on a predefined four-point ordinal scale from 0 to 3 according to the morphological criteria described below. The SM/MP ratio was assessed separately as a continuous morphometric variable.

#### 2.4.1. Lymphoid Nodules

• 0: absent;
• 1: 1–2 nodules;
• 2: 3–4 nodules;
• 3: 5 or more nodules.

Only distinct lymphoid nodular aggregates were counted. Diffuse lymphocytic infiltration without nodule formation was not included in the score.

#### 2.4.2. Fibrosis

• 0: absent;
• 1: mild focal collagen increase;
• 2: moderate multifocal collagen increase with stromal expansion;
• 3: severe diffuse collagen deposition with a sclerotic appearance.

Fibrosis was assessed primarily on H&E-stained sections; Masson’s trichrome staining was used to confirm collagen-rich areas and to support morphological assessment. Fibrosis scoring reflected the extent and distribution of collagen deposition and stromal expansion within the mucosal and submucosal compartments of the appendiceal wall. Masson’s trichrome staining was used as a qualitative confirmatory aid and was not subjected to quantitative collagen-area analysis.

#### 2.4.3. Luminal Status

• 0: normal open lumen;
• 1: mild narrowing;
• 2: marked narrowing;
• 3: severe narrowing or near-obliteration.

Luminal status was assessed only in well-oriented, non-tangential sections with intact wall architecture. Sections showing distortion, collapse, or artifacts suggestive of postmortem contraction or fixation effects were excluded from luminal scoring.

#### 2.4.4. Submucosal Adipose Accumulation

• 0: absent or minimal (≤10% of the submucosal area);
• 1: mild, small focal aggregates (>10–20%);
• 2: moderate, multifocal and partially coalescent adipose accumulation (>20–40%);
• 3: severe, diffuse or circumferential adipose accumulation (>40% of the submucosal area).

Only adipose tissue located within the submucosa was considered for scoring. Adipose tissue external to the muscularis propria was not included in the score. The percentage of submucosal adipose tissue was estimated visually by the observers across the evaluable submucosal area and assigned to the predefined percentage categories above. No automated image-segmentation or quantitative area-fraction analysis was performed.

### 2.5. Morphometric Assessment of the Submucosa-to-Muscularis Propria Ratio

The relative thickness of the submucosa and muscularis propria was assessed morphometrically using the submucosa-to-muscularis propria (SM/MP) ratio. Measurements were obtained from a representative, well-oriented, non-tangential H&E-stained section oriented perpendicular to the bowel wall to minimize overestimation associated with oblique sectioning.

Submucosal thickness (SM) was defined as the distance from the base of the mucosa to the inner border of the muscularis propria and included connective tissue, vessels, and adipose tissue within the submucosa. Muscularis propria thickness (MP) was defined as the total thickness of the muscularis propria, including the inner circular and outer longitudinal muscle layers.

**Figure 3:**
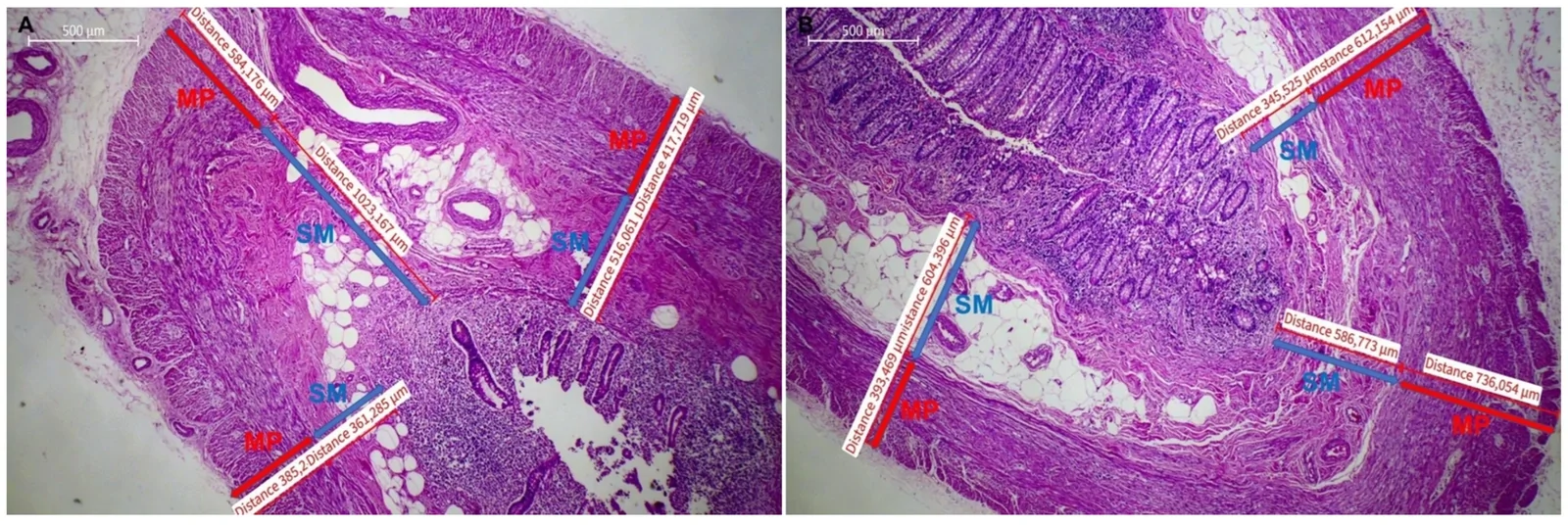
Morphometric measurement of the submucosa and muscularis propria in the human appendix. Representative H&E-stained sections showing paired measurements of submucosal (SM) and muscularis propria (MP) thicknesses at three non-overlapping regions within each appendiceal section. Measurements were performed along perpendicular transects in well-oriented areas of the appendiceal wall, avoiding prominent lymphoid nodules and tangentially sectioned regions. (**A**) Example demonstrating greater variation in submucosal thickness, with prominent submucosal adipose accumulation and marked fibrotic thickening of the SM layer. (**B**) Example with relatively thinner submucosal regions. For each case, the mean SM and mean MP values obtained from the three paired measurements were used to calculate the SM/MP ratio. The numerical distance values displayed on the images were generated automatically by ZEISS ZEN 3.10 software (Carl Zeiss Microscopy GmbH, Jena, Germany) after placement of the measurement lines. (H&E staining; scale bar = 500 µm).

Because submucosal thickness varied within individual sections, three non-overlapping regions corresponding to relatively thin, intermediate, and thick areas were selected from each representative section based on visual estimation of relative submucosal thickness. Areas containing prominent lymphoid nodules were excluded to minimize the potential effect of lymphoid tissue on submucosal thickness measurements. At each of the three measurement regions, submucosal and muscularis propria thicknesses were measured along the same perpendicular transect through the appendiceal wall. Thus, three paired SM and MP measurements were obtained for each case. The mean SM and mean MP values derived from these three transects were used to calculate the final SM/MP ratio. An annotated micrograph illustrating the SM/MP measurement protocol is provided in Figure 3.

The SM/MP ratio was calculated as:

SM/MP ratio = mean submucosal thickness/mean muscularis propria thickness

Morphometric measurements were performed using ZEISS ZEN 3.10 software (Carl Zeiss Microscopy GmbH, Jena, Germany) on digitized images of H&E-stained sections. Because selection of the three measurement regions involved visual judgment rather than systematic whole-slide or evenly spaced digital sampling, the SM/MP analysis was considered exploratory.

### 2.6. Statistical Analysis

Statistical analyses were performed using SPSS version 26.0 (IBM Corp., Armonk, NY, USA) and Python version 3.13.5. Standard proportional-odds ordinal logistic regression models were fitted using statsmodels version 0.14.6. Partial proportional-odds models were implemented in Python using a custom cumulative-logit maximum-likelihood approach with numerical optimization routines from SciPy version 1.17.0. Standard errors for the partial proportional-odds models were derived from the inverse observed Hessian matrix. Numerical stability of the custom models was assessed using alternative optimization algorithms, which yielded essentially identical coefficient estimates.

Continuous variables were summarized as mean ± standard deviation (SD), median with interquartile range (IQR), or range, as appropriate, and categorical variables were presented as frequencies and percentages. Distributional characteristics of continuous variables were assessed using the Shapiro–Wilk test together with visual inspection of Q–Q plots.

Because the histopathological scores were ordinal, sex-based comparisons of lymphoid nodules, fibrosis, luminal status, and submucosal adipose accumulation were performed using the Mann–Whitney U test. The SM/MP ratio showed evidence of deviation from normality and was therefore also compared between sexes using the Mann–Whitney U test.

Associations of age, body mass index (BMI), waist circumference, and abdominal subcutaneous fat thickness (ASFT) with the histopathological scores and SM/MP ratio were assessed using Spearman’s rank correlation coefficient (ρ).

Multivariable ordinal regression models were fitted separately for lymphoid nodule, fibrosis, luminal-status, and submucosal-adipose-accumulation scores. The primary models included age, sex, and BMI. Male sex was used as the reference category. For standard proportional-odds models, odds ratios (ORs) greater than 1 indicated higher odds of membership in a higher histopathological score category, whereas ORs below 1 indicated lower odds of a higher score. Results were reported as ORs with 95% confidence intervals (CIs) and two-sided *p* values.

The proportional-odds assumption was evaluated using a likelihood-ratio test of parallel lines. When evidence of violation was identified, partial proportional-odds models were fitted, allowing the coefficient responsible for the violation to vary across cumulative thresholds while retaining proportional effects for the remaining covariates. For lymphoid nodule scores, sex was modeled with threshold-specific coefficients, whereas age and BMI retained proportional effects.

Because only two cases had a fibrosis score of 0, fibrosis scores 0 and 1 were combined into an absent/mild category for multivariable regression to avoid unstable estimation at the lowest cumulative threshold. The resulting fibrosis outcome therefore comprised three ordered categories: absent/mild, moderate, and severe. In this model, age was allowed to have threshold-specific effects because the proportional-odds assumption was not satisfied for age, whereas sex and the adiposity measure retained proportional effects.

Standard proportional-odds models were retained for luminal status and submucosal adipose accumulation when the proportional-odds assumption was satisfied.

Because BMI, waist circumference, and ASFT represent related measures of adiposity and were substantially intercorrelated, they were not entered simultaneously into the same multivariable model. In addition to the primary age-, sex-, and BMI-adjusted models, separate age- and sex-adjusted models were fitted in which BMI, waist circumference, or ASFT was entered individually as the adiposity measure. Collinearity among the three adiposity measures was evaluated using pairwise correlations.

To evaluate the potential influence of postmortem interval (PMI) on the histopathological findings, additional sensitivity models included PMI together with age, sex, and BMI. Particular attention was given to luminal status because luminal configuration may be susceptible to postmortem change. Because only four cases had a completely open lumen, the normal-open and mild-narrowing categories were combined, yielding a three-level outcome comprising open/mild narrowing, marked narrowing, and severe narrowing/near-obliteration. To distinguish the effect of outcome recategorization from the effect of PMI adjustment, this three-level luminal model was first fitted with age, sex, and BMI without PMI and was subsequently refitted after adding PMI.

To assess potential confounding by cause of death, an additional sensitivity analysis was performed in which cause of death was dichotomized as natural versus non-natural death and included together with age, sex, and BMI. The same proportional-odds or partial proportional-odds specification used for each primary histopathological outcome was retained. Non-natural death was used as the reference category.

For descriptive purposes, cases were additionally stratified into three age groups (18–39, 40–59, and ≥60 years). These cut points were selected to provide interpretable younger-, middle-, and older-adult strata while retaining adequate numbers of cases in each group and were not intended to represent biological or diagnostic thresholds. Histopathological score distributions across age groups were compared using the Kruskal–Wallis test, and the distribution of natural versus non-natural deaths across age groups was compared using the chi-square test. These age-group analyses were exploratory, and age was retained as a continuous variable in the primary regression analyses.

Model adequacy was evaluated using likelihood-based fit indices, including the Akaike information criterion (AIC) and Bayesian information criterion (BIC), together with assessment of the proportional-odds assumption. Complete regression results were reported with the number of observations, covariate estimates, ORs, 95% CIs, and exact *p* values.

There were no missing data for the variables included in the present analyses; therefore, all 101 cases contributed to the correlation, morphometric, and regression analyses.

All statistical tests were two-tailed, and *p* < 0.05 was considered statistically significant. Because the study was exploratory and included multiple related comparisons, no formal correction for multiple testing was applied. Accordingly, statistically significant findings were interpreted cautiously, with emphasis on effect sizes, confidence intervals, consistency across models, and sensitivity analyses rather than on *p* values alone.

### 2.7. Reporting Guidelines

This study was conducted and reported in accordance with the STROBE (Strengthening the Reporting of Observational Studies in Epidemiology) guidelines for cross-sectional observational studies. A completed STROBE checklist and a study flow diagram summarizing case inclusion, exclusions, and the number of cases included in the final analyses are provided as Supplementary Materials.

### 2.8. Use of Generative AI Tools

During preparation of the manuscript, generative AI-assisted tools were used solely for language editing, translation, and improvement of textual clarity and organization. These tools were not used for data collection, histopathological assessment, statistical analysis, generation of study results, or scientific interpretation. All AI-assisted output was reviewed and edited by the authors, who take full responsibility for the final manuscript.

## 3. Results

### 3.1. Demographic, Anthropometric, and Forensic Characteristics

The final study population comprised 101 consecutive forensic autopsy cases, including 75 males (74.3%) and 26 females (25.7%). The mean age was 44.42 ± 18.47 years (range, 18–83 years). Mean BMI was 25.92 ± 4.54 kg/m^2^, mean waist circumference was 89.47 ± 14.29 cm, and mean ASFT was 3.77 ± 2.34 cm. The mean postmortem interval (PMI) was 5.07 ± 1.78 h, with a range of 2–9 h.

Cause-of-death information was available for all cases. The principal cause-of-death categories were asphyxia in 33 cases (32.7%), natural death in 32 (31.7%), trauma in 25 (24.8%), poisoning in 10 (9.9%), and other external causes in 1 (1.0%). The detailed distribution of specific causes of death is presented in Table 1. No data were missing for the demographic, anthropometric, PMI, or histopathological variables included in the analyses.

### 3.2. Histopathological Findings

**Figure 4:**
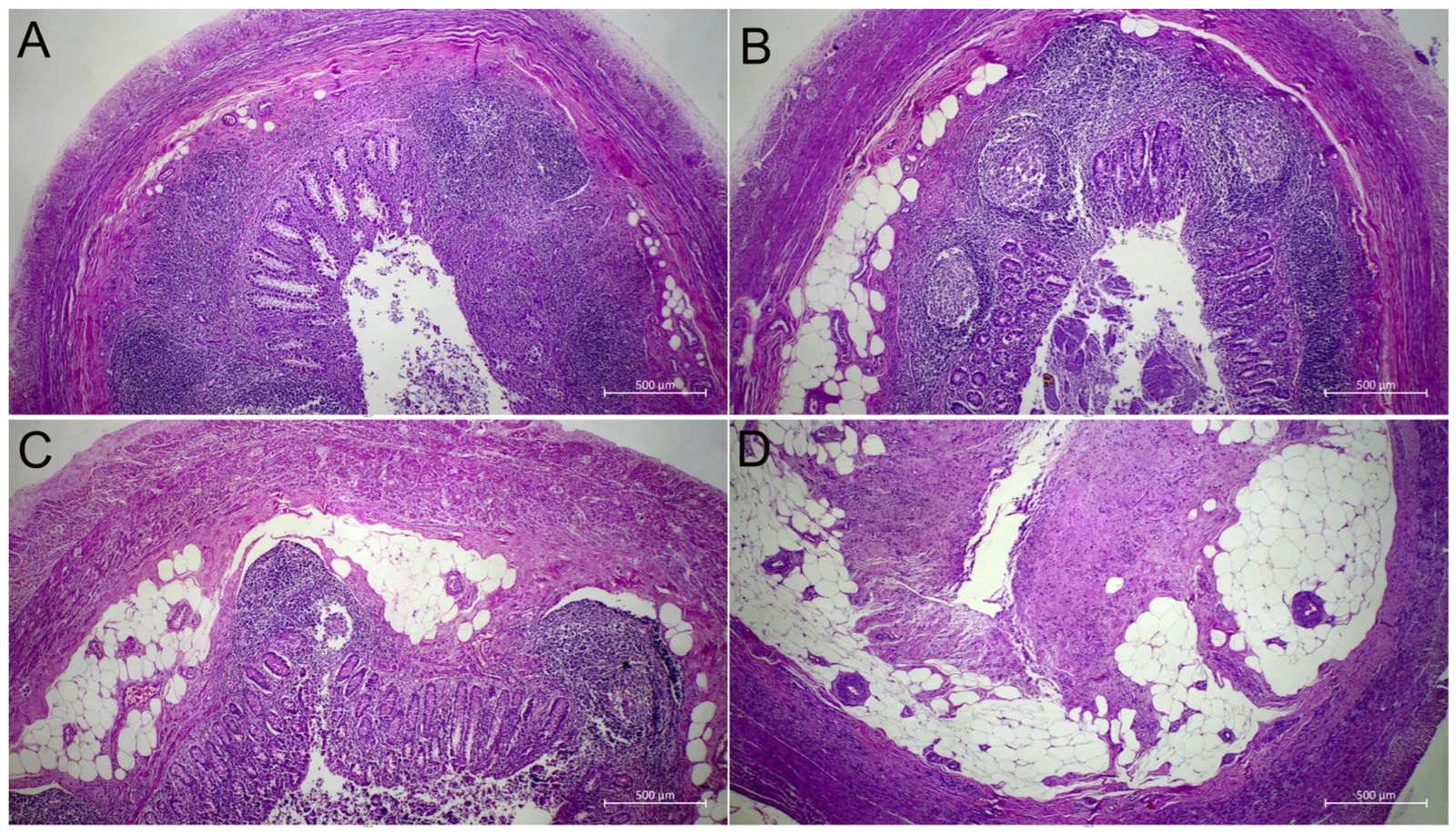
Histopathological spectrum of submucosal adipose accumulation in the appendix. Photomicrographs illustrating increasing submucosal adipose accumulation scores and associated changes in the appendiceal wall (H&E, ×40; scale bar = 500 µm). (**A**) Score 0: 19-year-old male (BMI 18.71 kg/m^2^; PMI 3 h; cause of death: gunshot wound to the chest). Preserved appendiceal wall architecture with prominent lymphoid nodules and no appreciable submucosal adipose accumulation; fibrous tissue is minimal. (**B**) Score 1: 29-year-old male (BMI 30.67 kg/m^2^; PMI 2 h; cause of death: sharp-force injury). Mild focal adipose accumulation with early collagen deposition and relatively preserved lymphoid tissue. (**C**) Score 2: 66-year-old female (BMI 38.30 kg/m^2^; PMI 4 h; cause of death: carbon monoxide poisoning). Moderate multifocal adipose expansion with partial lymphoid reduction and evident fibrosis. (**D**) Score 3: 82-year-old male (BMI 26.86 kg/m^2^; PMI 8 h; natural death due to cardiovascular disease). Severe diffuse adipose expansion with extensive fibrous tissue and marked lymphoid depletion.

**Figure 5:**
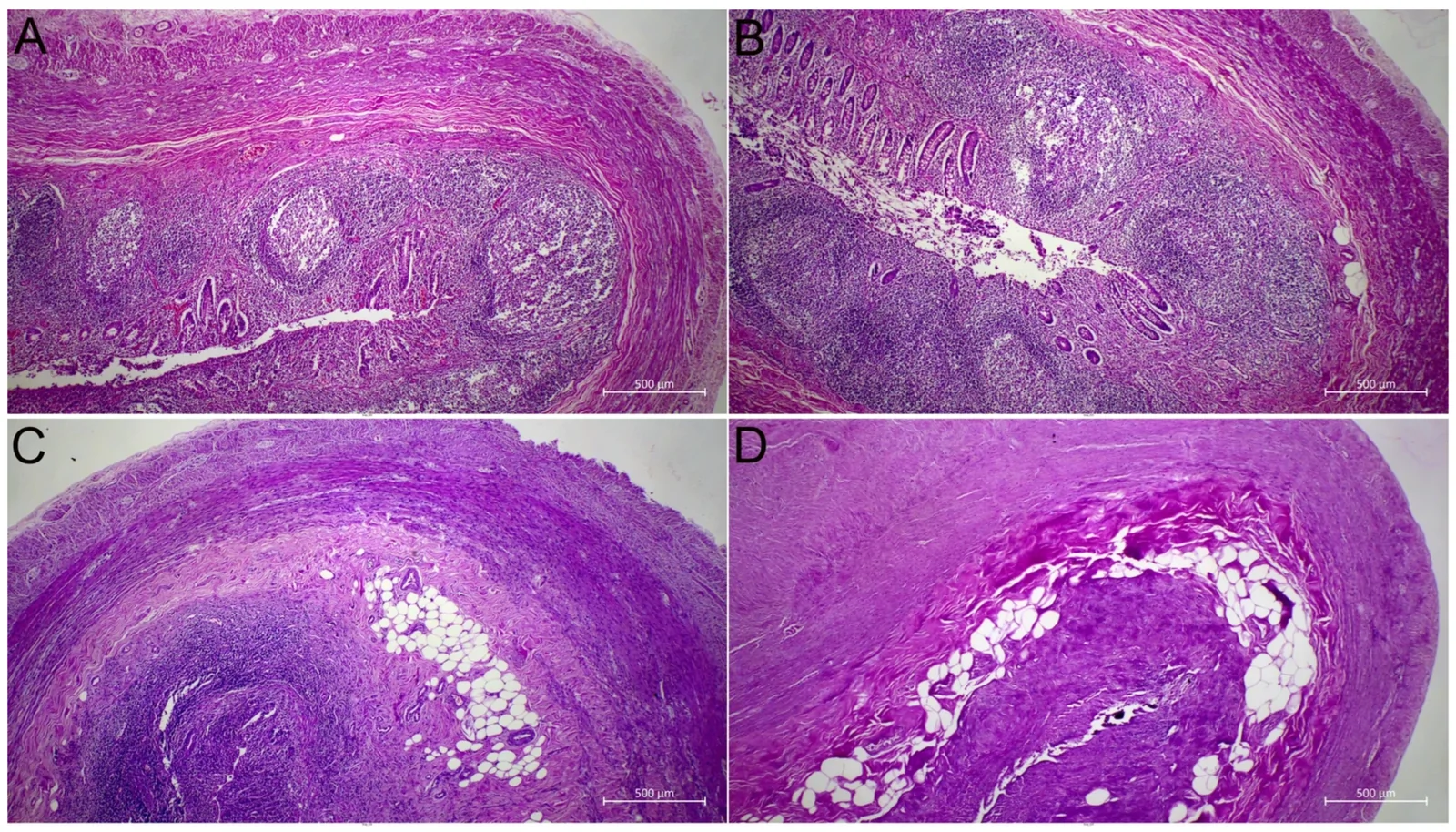
Histopathological spectrum of lymphoid, fibrous, and adipose changes in the appendix. Representative H&E photomicrographs illustrating histopathological variation in lymphoid tissue, fibrosis, and submucosal adipose accumulation within the appendiceal wall (H&E, ×40; scale bar = 500 µm). (**A**): 22-year-old male (BMI 19.38 kg/m^2^; PMI 3 h; cause of death: blunt head trauma). Lymphoid nodule score: 3; fibrosis score: 0; submucosal adipose accumulation score: 0. Preserved architecture with abundant lymphoid tissue and minimal fibrous or adipose components. (**B**): 39-year-old male (BMI 24.11 kg/m^2^; PMI 2 h; cause of death: gunshot wound). Lymphoid nodule score: 3; fibrosis score: 1; submucosal adipose accumulation score: 1. Mild increase in collagenous stroma and scattered submucosal adipocytes, with relatively preserved lymphoid tissue. (**C**): 62-year-old male (BMI 28.28 kg/m^2^; PMI 4 h; cause of death: hanging). Lymphoid nodule score: 1; fibrosis score: 2; submucosal adipose accumulation score: 2. Moderate fibrosis and submucosal adipose accumulation accompanied by partial lymphoid depletion. (**D**): 78-year-old male (BMI 32.53 kg/m^2^; PMI 7 h; natural death due to cardiovascular disease). Lymphoid nodule score: 0; fibrosis score: 3; submucosal adipose accumulation score: 3. Extensive collagenous stromal expansion and submucosal adipose accumulation with marked reduction in lymphoid tissue. These images illustrate compositional redistribution of the appendiceal wall components rather than establishing a specific mechanism of tissue compensation.

**Figure 6:**
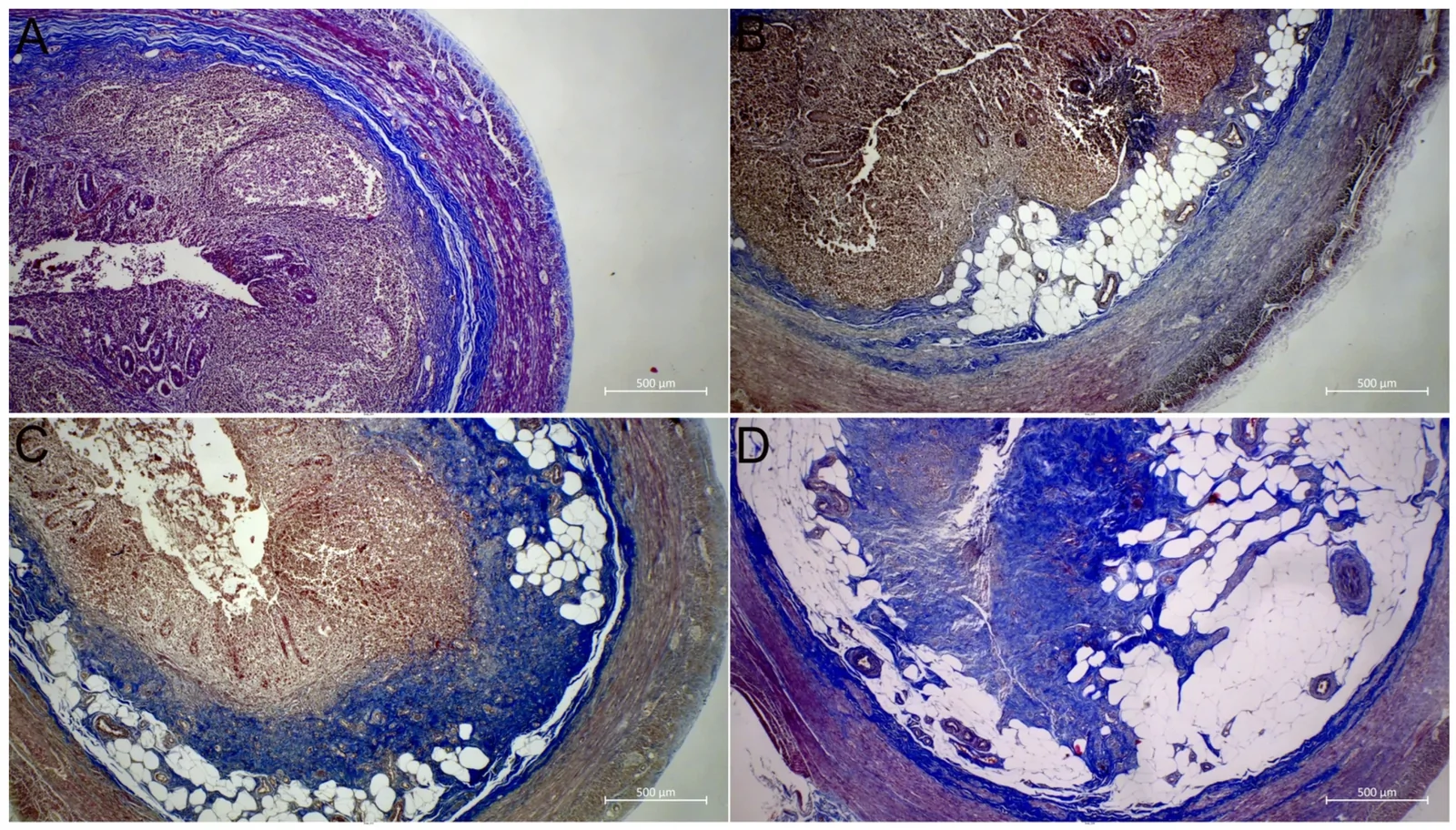
Masson’s trichrome staining demonstrating collagen-rich changes in the appendiceal wall. Representative Masson’s trichrome photomicrographs from cases with increasing H&E-based fibrosis scores, demonstrating collagen deposition and adipose accumulation within the appendiceal wall (Masson’s trichrome ×40; scale bar = 500 µm). (**A**) Score 0: 20-year-old male (BMI 18.92 kg/m^2^; PMI 3 h; cause of death: hanging). Preserved architecture with minimal collagen deposition and prominent lymphoid tissue. (**B**) Score 1: 36-year-old female (BMI 26.37 kg/m^2^; PMI 3 h; natural death due to cardiovascular disease). Mild focal adipose accumulation and early collagen deposition. (**C**) Score 2: 50-year-old male (BMI 26.70 kg/m^2^; PMI 6 h; cause of death: gunshot wound). Moderate adipose expansion with increased collagen deposition and partial lymphoid depletion. (**D**) Score 3: 69-year-old male (BMI 39.51 kg/m^2^; PMI 4 h; natural death due to cardiovascular disease). Extensive adipose and collagenous expansion with marked lymphoid reduction. Collagen fibers are highlighted in blue by Masson’s trichrome staining. Masson’s trichrome staining was used as a qualitative confirmatory aid in the assessment of fibrosis; collagen deposition was not quantified by digital image analysis or collagen-area fraction measurement.

The distribution of the histopathological scores is shown in Table 2. The mean SM/MP ratio was 1.18 ± 0.41 (range, 0.44–2.77). Submucosal adipose accumulation was observed in 90 of 101 cases (89.1%), although the severity varied considerably across the cohort. Representative examples of the histopathological spectrum of submucosal adipose accumulation, fibrosis, and lymphoid tissue reduction are shown in Figure 4, Figure 5 and Figure 6.

### 3.3. Sex-Related Differences

No statistically significant sex-based differences were observed in lymphoid nodule scores, fibrosis, luminal status, submucosal adipose accumulation, or the SM/MP ratio. Although female cases showed a numerically higher median lymphoid nodule score than male cases, this difference did not reach statistical significance (*p* = 0.096). Sex-based comparisons are summarized in Table 3.

### 3.4. Association with Age

Age was negatively correlated with lymphoid nodule scores (ρ = −0.301, *p* = 0.002) and positively correlated with fibrosis (ρ = 0.403, *p* < 0.001), luminal status (ρ = 0.228, *p* = 0.022), and submucosal adipose accumulation (ρ = 0.302, *p* = 0.002). No statistically significant correlation was observed between age and the SM/MP ratio (ρ = −0.138, *p* = 0.169).

### 3.5. Association with Adiposity Parameters

BMI showed a weak positive correlation with submucosal adipose accumulation (ρ = 0.196, *p* = 0.049), whereas its correlations with lymphoid nodule score, fibrosis, luminal status, and the SM/MP ratio were not statistically significant.

Waist circumference was not significantly correlated with any of the evaluated histopathological parameters or with the SM/MP ratio.

ASFT was positively correlated with luminal status (ρ = 0.218, *p* = 0.029) and submucosal adipose accumulation (ρ = 0.232, *p* = 0.020). No significant correlations were observed between ASFT and lymphoid nodule score, fibrosis, or the SM/MP ratio. The complete correlation results are presented in Table 4.

### 3.6. Multivariable Analysis

In the primary multivariable analyses, age, sex, and BMI were included as covariates. The proportional-odds assumption was satisfied for luminal status and submucosal adipose accumulation but was violated for lymphoid nodule and fibrosis scores. Accordingly, partial proportional-odds models were used for the latter outcomes. The primary multivariable regression results are summarized in Table 5.

For lymphoid nodule score, sex was modeled with threshold-specific coefficients, whereas age and BMI retained proportional effects. Increasing age was associated with lower lymphoid nodule scores (OR = 0.971, 95% CI 0.951–0.992, *p* = 0.007). BMI was not significantly associated with lymphoid nodule score (OR = 0.941, 95% CI 0.865–1.024, *p* = 0.159). The threshold-specific estimates for sex varied across the lymphoid score distribution and did not indicate a consistent directional association. The initial proportional-odds model showed evidence of non-proportionality (likelihood-ratio test, *p* = 0.006), whereas the partial proportional-odds specification adequately accounted for this violation (*p* = 0.205).

For fibrosis, scores 0 and 1 were combined because only two cases had a score of 0, resulting in three ordered categories: absent/mild, moderate, and severe. Age showed threshold-specific associations with fibrosis. Each additional year of age was associated with higher odds of moderate/severe versus absent/mild fibrosis (OR = 1.039, 95% CI 1.013–1.065, *p* = 0.003) and with higher odds of severe versus absent/mild/moderate fibrosis (OR = 1.087, 95% CI 1.050–1.125, *p* < 0.001). Neither female sex (OR = 1.340, 95% CI 0.546–3.284, *p* = 0.523) nor BMI (OR = 0.921, 95% CI 0.841–1.009, *p* = 0.077) was significantly associated with fibrosis. The proportional-odds assumption was violated in the three-level model (*p* = 0.039), whereas the partial proportional-odds model with threshold-specific age effects showed no remaining evidence of non-proportionality (*p* = 0.691).

For luminal status, increasing age was associated with higher narrowing scores in the primary four-level model (OR = 1.022, 95% CI 1.001–1.044, *p* = 0.038), whereas sex and BMI were not significantly associated with the outcome. For submucosal adipose accumulation, increasing age was also associated with higher scores (OR = 1.027, 95% CI 1.007–1.048, *p* = 0.010). BMI showed a positive but non-significant adjusted association with submucosal adipose accumulation (OR = 1.076, 95% CI 0.987–1.173, *p* = 0.095), and sex was not significantly associated with this outcome. The proportional-odds assumption was satisfied for both luminal status (*p* = 0.300) and submucosal adipose accumulation (*p* = 0.274).

Because BMI, waist circumference, and ASFT were strongly intercorrelated, they were evaluated in separate age- and sex-adjusted models rather than entered simultaneously. Pairwise Spearman correlations were ρ = 0.800 for BMI and waist circumference, ρ = 0.854 for BMI and ASFT, and ρ = 0.749 for waist circumference and ASFT. In these separate models, waist circumference was not significantly associated with any histopathological outcome. In contrast, ASFT was associated with higher luminal-status scores (OR = 1.212, 95% CI 1.027–1.431, *p* = 0.023) and higher submucosal-adipose-accumulation scores (OR = 1.203, 95% CI 1.022–1.416, *p* = 0.026). Separate age- and sex-adjusted models examining BMI, waist circumference, and ASFT are summarized in Table 6. Complete covariate estimates and model-fit statistics for these adiposity models are provided in Supplementary Table S1.

In sensitivity analyses incorporating PMI, PMI was not significantly associated with the evaluated histopathological outcomes. For luminal status, the sparse normal-open category was combined with mild narrowing, yielding a three-level outcome. To distinguish the effect of outcome recategorization from that of PMI adjustment, the three-level model was first fitted without PMI. In this model, the association between age and luminal status was OR = 1.021 (95% CI 1.000–1.042, *p* = 0.051). After PMI was added, the age point estimate remained essentially unchanged (OR = 1.021, 95% CI 0.999–1.044, *p* = 0.057), while PMI was not significantly associated with luminal status (OR = 0.989, 95% CI 0.789–1.238, *p* = 0.921). Thus, the weaker statistical evidence relative to the primary four-level model cannot be attributed specifically to PMI adjustment, because recategorization of the luminal outcome also affected the model. PMI-adjusted sensitivity analyses are summarized in Table 7, with complete covariate estimates and model-fit statistics provided in Supplementary Table S2.

Cause of death was examined as a potential confounder by dichotomizing cases as natural (*n* = 32) or non-natural (*n* = 69) deaths, with non-natural death used as the reference category. Natural deaths were more frequent in older age groups, accounting for 14.3% of cases aged 18–39 years, 34.5% of those aged 40–59 years, and 65.2% of those aged ≥60 years (χ^2^ = 18.91, *p* < 0.001). After additional adjustment for cause of death, natural death was not independently associated with lymphoid nodule score (OR = 0.542, 95% CI 0.222–1.323, *p* = 0.179), fibrosis (OR = 1.160, 95% CI 0.458–2.940, *p* = 0.754), luminal status (OR = 1.228, 95% CI 0.488–3.091, *p* = 0.663), or submucosal adipose accumulation (OR = 0.905, 95% CI 0.385–2.125, *p* = 0.818). The age associations with fibrosis remained statistically significant at both severity thresholds (OR = 1.037, 95% CI 1.009–1.065, *p* = 0.009; and OR = 1.085, 95% CI 1.046–1.125, *p* < 0.001), as did the association with submucosal adipose accumulation (OR = 1.028, 95% CI 1.006–1.052, *p* = 0.015). In contrast, the age associations with lymphoid nodule score (OR = 0.978, 95% CI 0.955–1.001, *p* = 0.063) and luminal status (OR = 1.020, 95% CI 0.996–1.044, *p* = 0.109) no longer reached conventional statistical significance. Complete covariate estimates and model-fit statistics for the cause-of-death sensitivity models are provided in Supplementary Table S3.

### 3.7. Distribution of Histopathological Scores Across Age Groups

For descriptive purposes, histopathological score distributions were additionally examined across three age groups (18–39, 40–59, and ≥60 years) (Table 8). Lymphoid nodule scores decreased across age groups, whereas fibrosis and submucosal adipose accumulation scores shifted toward higher categories with increasing age. The most pronounced pattern was observed for fibrosis, with severe fibrosis present in 60.9% of cases aged ≥60 years compared with 4.1% of those aged 18–39 years. Luminal-status distributions did not differ in the omnibus Kruskal–Wallis test across the three age groups (*p* = 0.312), although a positive monotonic association with continuous age was observed (ρ = 0.228, *p* = 0.022).

## 4. Discussion

The present prospective cross-sectional forensic autopsy study identified distinct age- and adiposity-associated patterns in appendiceal histology. Advancing age was associated with lower lymphoid nodule scores and higher fibrosis, luminal-status, and submucosal-adipose-accumulation scores in unadjusted analyses. In the primary multivariable analyses, age remained associated with lower lymphoid nodule scores, and fibrosis showed threshold-dependent age associations. Age was also associated with higher luminal-status and submucosal-adipose-accumulation scores in the primary proportional-odds models. After additional adjustment for natural versus non-natural death, the age associations with fibrosis and submucosal adipose accumulation persisted, whereas those with lymphoid nodule score and luminal status no longer reached conventional statistical significance. In the three-level luminal sensitivity analysis, the age estimate was essentially unchanged after adding PMI (OR = 1.021 before and after adjustment), while the statistical evidence was weaker than in the primary four-level model (*p* = 0.051 without PMI and *p* = 0.057 with PMI). Among the adiposity measures, BMI showed only a weak unadjusted association with submucosal adipose accumulation, whereas waist circumference showed no significant bivariate associations. ASFT was associated with both luminal status and submucosal adipose accumulation in unadjusted and age- and sex-adjusted analyses. The SM/MP ratio showed no significant association with age or any adiposity measure. No consistent sex-related differences were identified. Taken together, these findings indicate that appendiceal histological variation across adulthood is heterogeneous and should not be interpreted as a single uniform remodeling process.

### 4.1. Age-Related Changes

The inverse association between age and lymphoid nodule score is consistent with previous and recent human studies demonstrating progressive involution of appendiceal lymphoid tissue with advancing age [3,4,5]. Sumi et al. reported a marked decline in the number of lymphoid follicles across increasing age groups [4], while a recent small study by Quien et al. reported more frequent lymphoid follicle reduction in aged appendiceal samples [5]. In the present cohort, age was negatively correlated with lymphoid nodule score (ρ = −0.301, *p* = 0.002), and this association remained evident after adjustment for sex and BMI (OR = 0.971, 95% CI 0.951–0.992, *p* = 0.007). These findings are also compatible with the broader concept of immunosenescence, although the present study did not investigate cellular or molecular mechanisms of immune aging [15]. Collectively, the available evidence supports the interpretation that reduced lymphoid prominence may constitute part of the background histological variation encountered with advancing age rather than, in isolation, evidence of a specific pathological process. Nevertheless, because the present study was cross-sectional, the temporal course of lymphoid involution within individuals cannot be determined.

Age was also positively correlated with fibrosis (ρ = 0.403, *p* < 0.001). The existing literature on age-associated appendiceal fibrosis is not entirely consistent. In a histopathological study including 101 autopsy appendices and more than 3000 surgically resected appendices, Andreou et al. observed fibrosis more frequently in the older autopsy group and suggested that it may represent an age-related change, while acknowledging that previous inflammation could contribute to this finding [16]. The same caveat applies to the present cohort: although cases with active appendiceal inflammation and specific appendiceal disease were excluded, remote or resolved inflammatory episodes could not be reliably ruled out and may have contributed to fibrosis in some cases. In contrast, Takeuchi did not identify a clear relationship between aging and appendiceal fibrosis [3]. More recently, Quien et al. described fibrous obliteration of the appendiceal tip in 71–75% of adult and geriatric samples versus 20% of pediatric samples, although the difference was not statistically significant in that small cohort (*n* = 20); moreover, tip fibrous obliteration should not be equated with the appendiceal wall fibrosis scored in the present study [5]. In our cohort, the proportional-odds assumption was not satisfied for fibrosis. After fibrosis scores 0 and 1 were combined because only two cases had a score of 0, age demonstrated threshold-specific associations. Each additional year of age was associated with greater odds of moderate/severe versus absent/mild fibrosis (OR = 1.039, 95% CI 1.013–1.065, *p* = 0.003) and with greater odds of severe versus absent/mild/moderate fibrosis (OR = 1.087, 95% CI 1.050–1.125, *p* < 0.001). The point estimate for the age association was higher at the severe fibrosis threshold, although no formal comparison between threshold-specific effect estimates was performed. These findings support an age-associated pattern of appendiceal fibrosis but do not establish aging as a direct biological cause of fibrosis.

Luminal status also showed a positive correlation with age (ρ = 0.228, *p* = 0.022). In the primary age-, sex-, and BMI-adjusted four-level model, increasing age was associated with higher luminal-narrowing scores (OR = 1.022, 95% CI 1.001–1.044, *p* = 0.038). However, interpretation of this finding requires particular caution. In the three-level sensitivity analysis, the age point estimate was essentially unchanged after the addition of PMI (OR = 1.021 both before and after PMI adjustment), while the *p* value changed from 0.051 to 0.057. Therefore, the weaker statistical evidence relative to the primary four-level model cannot be attributed specifically to PMI adjustment, because the luminal outcome was also recategorized. PMI itself was not significantly associated with luminal status within the observed 2–9 h range (OR = 0.989, 95% CI 0.789–1.238, *p* = 0.921). Given the relatively narrow PMI distribution, the absence of a statistically significant PMI association should not be interpreted as evidence that postmortem interval has no influence on luminal configuration. Residual postmortem and tissue-processing effects therefore cannot be excluded.

Appendiceal morphology and diameter remain important components of the contemporary diagnostic assessment of suspected acute appendicitis, with current guidelines emphasizing imaging within an integrated clinical diagnostic approach [17,18]. However, the present study did not include matched antemortem imaging, and histological luminal narrowing should not be considered equivalent to imaging-based appendiceal diameter. Accordingly, no diagnostic imaging threshold can be derived from the present findings. Future studies integrating antemortem radiological findings with postmortem histopathology would be valuable for determining whether the structural patterns observed histologically have corresponding imaging features.

Finally, age was positively correlated with submucosal adipose accumulation (ρ = 0.302, *p* = 0.002), and this association remained statistically significant in the primary adjusted model (OR = 1.027, 95% CI 1.007–1.048, *p* = 0.010). A small descriptive report suggested that aging may induce steatotic changes in the appendix [3], and contemporary evidence indicates that adipose tissue undergoes substantial age- and depot-dependent remodeling [9], providing biological context for the present observation. However, no available evidence establishes that the microscopic submucosal adipose accumulation observed in the appendix represents the same process as age-related remodeling in established systemic adipose depots. Because the present study did not assess metabolic status, visceral adiposity, adipocyte biology, or molecular mechanisms, the biological basis of this association cannot be determined. It should therefore be interpreted as an age-associated morphological pattern rather than evidence of a defined mechanistic process.

Cause of death was also considered as a potential confounder. After additional adjustment for natural versus non-natural death, cause of death itself was not independently associated with any of the evaluated histopathological outcomes. The age associations with fibrosis and submucosal adipose accumulation remained statistically significant, whereas those with lymphoid nodule score and luminal status no longer reached conventional statistical significance. These findings indicate that the age associations with lymphoid depletion and luminal narrowing were less robust to additional adjustment for cause of death and should therefore be interpreted more cautiously.

### 4.2. Adiposity and Submucosal Adipose Accumulation

The associations between adiposity measures and appendiceal histology were modest and varied according to the anthropometric measure examined. BMI showed a weak positive correlation with submucosal adipose accumulation (ρ = 0.196, *p* = 0.049); however, this association was no longer statistically significant after adjustment for age and sex (OR = 1.076, 95% CI 0.987–1.173, *p* = 0.095). Waist circumference was not significantly correlated with submucosal adipose accumulation or with the other histopathological outcomes and likewise showed no significant adjusted association with submucosal adipose accumulation (OR = 1.012, 95% CI 0.985–1.040, *p* = 0.379). BMI and waist circumference are widely used anthropometric indicators of overall and central adiposity, respectively [19]; however, different anthropometric measures capture distinct aspects of body-fat distribution and should not be regarded as interchangeable indicators of specific adipose-tissue compartments [20]. Accordingly, the lack of a strong association between BMI or waist circumference and microscopic submucosal adipose accumulation in the appendix is not unexpected and suggests that these conventional anthropometric measures may not closely reflect local appendiceal adipose deposition.

In contrast, ASFT showed positive correlations with both luminal status (ρ = 0.218, *p* = 0.029) and submucosal adipose accumulation (ρ = 0.232, *p* = 0.020). These associations remained statistically significant in separate age- and sex-adjusted models, with greater ASFT associated with higher luminal-status scores (OR = 1.212, 95% CI 1.027–1.431, *p* = 0.023) and higher submucosal-adipose-accumulation scores (OR = 1.203, 95% CI 1.022–1.416, *p* = 0.026). Regional abdominal subcutaneous adipose-tissue thickness has been shown to vary in relation to anthropometric and clinical characteristics [10]. Nevertheless, the ASFT measurement used in the present study represents abdominal subcutaneous adiposity and is not a direct measure of visceral or appendiceal fat. The observed associations should therefore not be interpreted as evidence that systemic or regional adiposity directly causes adipose accumulation or luminal change within the appendiceal wall.

Submucosal adipose accumulation was present to some degree in 90 of 101 cases (89.1%). This high frequency should not be equated with appendiceal or ileocecal lipomatosis. Clinically reported lipomatosis represents a distinct pathological condition characterized by prominent submucosal adipose infiltration and may occasionally mimic or accompany acute appendiceal disease [21,22]. In contrast, the present study assessed microscopic submucosal adipose accumulation using predefined semi-quantitative categories in appendices obtained without a clinical indication for appendectomy and after exclusion of specific appendiceal pathology. The frequent occurrence of low-to-moderate grades in this forensic autopsy cohort therefore suggests that some degree of submucosal adipose tissue may occur as part of the morphological spectrum encountered in adult appendices; however, the present data do not establish a threshold separating background variation from pathological lipomatosis.

Adipose accumulation associated with inflammatory bowel disease represents another distinct phenomenon. In Crohn’s disease, creeping fat consists primarily of remodeled mesenteric adipose tissue surrounding inflamed bowel and is associated with inflammatory and fibrotic processes [23]. The present cohort excluded cases with marked active inflammation or histological evidence of inflammatory bowel disease involving the appendix. Moreover, the adipose tissue evaluated in this study was located within the appendiceal submucosa rather than in the mesenteric compartment. The observed submucosal adipose accumulation should therefore not be interpreted as analogous to Crohn-related creeping fat.

Taken together, these findings suggest that the relationship between body habitus and appendiceal histology is not uniform. BMI showed only a weak unadjusted association, waist circumference showed no significant association, whereas ASFT demonstrated modest adjusted associations with luminal narrowing and submucosal adipose accumulation. The stronger association observed with a regional subcutaneous measure than with BMI or waist circumference may indicate that different adiposity measures capture distinct aspects of body-fat distribution; however, the present study cannot determine whether this pattern reflects a direct biological relationship with appendiceal adipose deposition. These findings should therefore be regarded as exploratory and require confirmation using direct measures of visceral and regional adipose tissue in independent cohorts.

### 4.3. SM/MP Ratio

The SM/MP ratio showed no significant association with age (ρ = −0.138, *p* = 0.169), BMI (ρ = 0.048, *p* = 0.637), waist circumference (ρ = 0.052, *p* = 0.609), or ASFT (ρ = 0.076, *p* = 0.449). The mean SM/MP ratio was 1.18 ± 0.41, with values ranging from 0.44 to 2.77. Thus, unlike the semi-quantitative lymphoid, fibrotic, luminal, and adipose features, the relative thickness of the submucosa and muscularis propria did not demonstrate a detectable relationship with age or the evaluated measures of adiposity in this cohort.

These findings should not be interpreted as evidence that the absolute thickness or composition of the appendiceal wall remains unchanged across adulthood. The SM/MP ratio is a relative measure; proportional changes in both layers could occur without producing a substantial change in the ratio. Moreover, changes in lymphoid tissue, fibrosis, vascular components, or submucosal adipose tissue may alter tissue composition without necessarily producing a corresponding change in the overall SM/MP ratio.

The morphometric findings should also be regarded as exploratory. Measurements were obtained from three visually selected, well-oriented regions representing relatively thin, intermediate, and thick portions of the wall, rather than from systematically sampled or whole-slide-derived locations. Although the submucosa and muscularis propria were measured along the same perpendicular transect at each region, separate intra- or inter-observer reliability data were not available for these morphometric measurements. Consequently, subtle associations may have remained undetected, and the absence of statistically significant correlations should not be interpreted as proof of morphometric invariance.

Future studies using systematic whole-slide sampling, digital morphometry, and independently validated measurement reproducibility may clarify whether age- or adiposity-related alterations in absolute wall-layer thickness occur despite the absence of detectable differences in the SM/MP ratio in the present study.

### 4.4. Forensic and Medico-Legal Relevance

The forensic relevance of the present findings lies primarily in providing descriptive histological context rather than in establishing a new diagnostic test or a routine indication for appendiceal sampling. Histopathology can contribute important information in medico-legal autopsies, particularly when gross examination alone does not adequately clarify a suspected pathological process [2,24]; however, its diagnostic contribution is case-dependent and varies across categories of death [2,25]. The appendix is rarely the principal focus of a forensic examination, and autopsy-practice data indicate that its histological examination may be selective, particularly when gross abnormalities are present [1]. When appendiceal tissue is nevertheless examined histologically, knowledge of the range of lymphoid, fibrotic, luminal, and adipose findings encountered in specimens obtained without a clinical indication for appendectomy may provide useful context for interpretation. The age-stratified distributions presented in Table 8 provide additional descriptive context for the spectrum of appendiceal histological findings across adulthood; however, these groups were exploratory, relatively small—particularly the ≥60-year group (*n* = 23)—and were not population-based reference strata.

Contemporary surgical series and pathology reviews have substantially expanded the recognized spectrum of incidental appendiceal abnormalities [11,12,13]. However, such material is predominantly derived from appendectomy specimens selected because of clinical suspicion or another surgical indication and therefore represents a different observational setting from forensic autopsy tissue. Earlier histopathological work incorporating autopsy specimens also demonstrated substantial appendiceal variation outside the setting of acute appendicitis, including a greater frequency of fibrosis in older autopsy cases [16]. The present cohort extends this observational context by systematically examining age- and adiposity-associated variation in consecutive forensic autopsy specimens not selected for appendectomy. These findings may assist in contextualizing an incidental microscopic observation, but they do not define a boundary between physiological and pathological change.

Luminal narrowing requires particular caution. Although age was associated with higher luminal-status scores in the primary four-level model, the statistical evidence was weaker in the three-level sensitivity analysis irrespective of PMI adjustment (OR = 1.021; *p* = 0.051 without PMI and *p* = 0.057 with PMI). PMI itself was not significantly associated with luminal status within the observed 2–9 h range. Nevertheless, the absence of a statistically significant PMI association does not exclude postmortem influences on tissue configuration. Accordingly, luminal narrowing observed in postmortem histological sections should be interpreted together with macroscopic autopsy findings, histological evidence of inflammation or other disease, available clinical information, and the overall circumstances of death rather than as an isolated indicator of antemortem appendiceal pathology.

Importantly, the present study did not include comparator groups with acute appendicitis, inflammatory bowel disease, chronic obliterative appendiceal disease, or experimentally defined postmortem artifact. It also did not assess reader misclassification, diagnostic sensitivity or specificity, or whether appendiceal histology altered cause-of-death determination. This distinction is particularly important because the reported contribution of histology to cause-of-death assessment varies according to case selection and death category in medico-legal practice [2,25]. Therefore, the present data cannot demonstrate that the observed appendiceal patterns distinguish background variation from disease with diagnostic accuracy, nor do they support routine histological examination of the appendix in all forensic autopsies. No diagnostic threshold for fibrosis, luminal narrowing, lymphoid depletion, or submucosal adipose accumulation can be derived from this study.

The practical contribution of the present study is therefore deliberately limited and descriptive. When appendiceal tissue is examined histologically in a medico-legal case, awareness that substantial variation in lymphoid, fibrotic, luminal, and adipose features may occur across adult forensic autopsy cases can provide additional morphological context for interpretation. Such findings should always be integrated with the complete autopsy examination, clinical and circumstantial information, and other relevant ancillary investigations and should not, in isolation, be regarded as evidence of antemortem gastrointestinal disease or as a contributor to the cause or manner of death.

### 4.5. Sex-Related Findings

No consistent sex-related differences were identified across the histopathological features evaluated in the present cohort. In unadjusted comparisons, lymphoid nodule score, fibrosis, luminal status, submucosal adipose accumulation, and the SM/MP ratio did not differ significantly between male and female cases. Although female cases showed a numerically higher median lymphoid nodule score than male cases [2 (IQR 0–2) vs. 1 (IQR 0–1)], this difference did not reach statistical significance (*p* = 0.096).

In the partial proportional-odds model for lymphoid nodule score, the effect of sex varied across cumulative thresholds. Female sex was not significantly associated with having a score > 0 versus 0 (OR = 0.827, 95% CI 0.308–2.219, *p* = 0.705) or with having a score of 3 versus ≤2 (OR = 1.652, 95% CI 0.359–7.610, *p* = 0.519). However, female sex was associated with higher odds of having a lymphoid nodule score > 1 versus ≤1 (OR = 4.401, 95% CI 1.627–11.907, *p* = 0.004). Because this association was confined to a single cumulative threshold and the other threshold-specific estimates were not statistically significant, the findings do not indicate a consistent overall sex-related pattern across the lymphoid score distribution.

Sex was not significantly associated with fibrosis, luminal status, or submucosal adipose accumulation in the multivariable models. These findings should nevertheless be interpreted cautiously because the cohort was predominantly male (75/101, 74.3%), with only 26 female cases. This imbalance limits the precision of sex-specific estimates and reduces the ability to detect smaller or more complex sex-related differences. Larger cohorts with a more balanced sex distribution would be required to determine whether reproducible sex-related variation exists in appendiceal histology.

### 4.6. Strengths and Limitations

Several strengths of the present study should be acknowledged. The study used a prospective cross-sectional design with consecutive case inclusion and a standardized protocol for anthropometric measurements, appendiceal sampling, histological processing, and semi-quantitative assessment. All 101 cases had complete data for the variables included in the analyses. Histopathological scoring was performed independently by two experienced observers blinded to demographic and anthropometric information, followed by blinded pathologist review and consensus assessment when required. Inter-observer agreement was substantial to excellent across the four scored histopathological parameters. In addition, the statistical approach accounted for violations of the proportional-odds assumption, evaluated BMI, waist circumference, and ASFT in separate age- and sex-adjusted models, and incorporated PMI and cause of death in sensitivity analyses.

Several limitations should also be considered. First, this was a single-center forensic autopsy study based on 101 consecutive eligible cases within a defined study period, and no formal a priori sample-size calculation was performed. The predominance of male cases (74.3%) limits the precision of sex-specific estimates. Although additional adjustment for natural versus non-natural cause of death did not identify an independent association with the evaluated histopathological outcomes, cause of death was strongly related to age, and detailed data on manner of death, chronic comorbidities, medication exposure, and agonal circumstances were not systematically available. Residual confounding from underlying disease or circumstances surrounding death therefore cannot be excluded. Diabetes status was determined from documented medical history and available medical records; therefore, the possibility of unrecognized or undocumented diabetes cannot be completely excluded.

Second, despite the relatively short PMI observed in this cohort (2–9 h), postmortem effects cannot be fully eliminated. All bodies were handled according to the standard refrigeration protocol of the forensic autopsy unit; however, case-specific refrigeration parameters were not separately recorded. Information on early autolytic changes, resuscitation, fluid shifts, and body cooling before arrival at the autopsy unit was not systematically recorded. These factors may influence tissue configuration, particularly luminal appearance, and residual postmortem confounding cannot therefore be completely excluded. In some cases, the time of death recorded in the medico-legal file was estimated from available clinical, scene, or investigative information rather than directly observed, introducing some unavoidable uncertainty into PMI measurement. In addition, the relatively narrow PMI range (2–9 h; SD 1.78 h) limited the ability to assess PMI-related variation. Because cases with advanced appendiceal putrefaction or autolysis precluding reliable histopathological evaluation were excluded, the analytical cohort may also underrepresent cases with longer or less favorable postmortem conditions.

Third, histopathological assessment was based on one representative full-thickness mid-appendiceal tissue block per case. Although three H&E sections and one Masson’s trichrome section were examined to improve tissue representation, lymphoid nodule counts were not normalized to total mucosal length or tissue area. Therefore, residual sampling variability cannot be excluded. Similarly, fibrosis and submucosal adipose accumulation were assessed semi-quantitatively rather than by automated digital area measurement. Although inter-observer agreement was high, semi-quantitative scoring remains inherently dependent on observer interpretation. Furthermore, although cases with acute appendicitis, marked active inflammation, inflammatory bowel disease involving the appendix, and other specific appendiceal pathology were excluded, remote or resolved appendiceal inflammation could not be systematically identified or excluded. Consequently, some fibrotic or luminal changes may partly reflect previous inflammatory injury rather than age-associated variation alone.

The SM/MP analysis has additional limitations. Measurement regions were visually selected from well-oriented sections and were not obtained using systematic evenly spaced sampling or whole-slide digital morphometry. Separate intra- or inter-observer reliability data were not available for the morphometric measurements. Accordingly, the SM/MP findings should be regarded as exploratory, and the absence of significant associations should not be interpreted as evidence of true morphometric invariance.

Anthropometric limitations should also be acknowledged. ASFT reflects subcutaneous rather than visceral adiposity, and no direct measure of visceral adipose tissue was available. Replicate anthropometric measurements were not separately documented in the study records; therefore, repeat-measurement reliability could not be assessed. Stature was measured in the supine position using a measuring tape rather than by conventional standing stadiometry, which may have introduced additional measurement variability into BMI estimation. In addition, the present study did not include matched antemortem imaging or comparator groups with acute appendicitis, inflammatory bowel disease, chronic obliterative disease, or defined postmortem artifact. Consequently, diagnostic accuracy, disease discrimination, or clinically applicable thresholds cannot be derived from these data. Finally, multiple exploratory analyses were performed without formal correction for multiplicity; therefore, particularly modest associations close to the conventional significance threshold should be interpreted cautiously and require confirmation in independent cohorts.

### 4.7. Contribution to the Literature and Future Directions

The present study contributes to the comparatively limited literature characterizing appendiceal histology in specimens obtained outside the setting of clinically indicated appendectomy. Previous autopsy and human histological investigations have documented age-related lymphoid and structural variation in the appendix [3,4,5,16], whereas recent large surgical series have substantially expanded knowledge of the broader histopathological spectrum encountered in appendectomy specimens [13]. The present study addresses a different question by systematically evaluating lymphoid nodules, fibrosis, luminal status, submucosal adipose accumulation, and the SM/MP ratio together with demographic, anthropometric, and postmortem variables in a consecutive forensic autopsy cohort. The findings indicate that these histological features do not vary uniformly: age showed its most consistent associations with fibrotic and adipose features, which persisted after additional adjustment for cause of death, whereas associations with lymphoid and luminal features were less robust; associations with adiposity were more limited and were most evident for ASFT in relation to luminal status and submucosal adipose accumulation.

Further studies should prioritize multicenter forensic autopsy cohorts with larger and more sex-balanced samples, systematic documentation of relevant comorbidities and manner of death, and detailed control of postmortem factors such as refrigeration, pre-fixation interval, and autolytic change. Histological assessment could be strengthened by normalization of lymphoid counts to tissue area or mucosal length, systematic whole-slide digital morphometry, quantitative assessment of collagen and adipose tissue, and formal reproducibility testing of morphometric measurements. Comparator groups with acute appendicitis, chronic obliterative changes, and inflammatory bowel disease would be particularly important because disease-related appendiceal remodeling may include inflammatory, fibrotic, and obliterative changes that overlap morphologically with some of the features assessed in the present study. Recent observations in ulcerative colitis, for example, demonstrate time-dependent appendiceal remodeling from active inflammation toward fibrous obliteration [26]. Direct comparison with such disease groups would be required before the diagnostic discriminatory value of the present histological patterns could be evaluated.

Matched antemortem imaging and postmortem histopathology represent another particularly relevant direction for future research. Contemporary guidelines emphasize imaging as an important component of the evaluation of suspected appendicitis [17,18]. The importance of integrating imaging findings with the broader clinical and anatomical context is also evident in diagnostically challenging presentations such as appendicitis in situs viscerum inversus [27]. Direct comparison of antemortem ultrasonographic or CT findings with postmortem gross and histological measurements in the same individuals could help determine whether histological luminal changes have identifiable radiological correlates and clarify which findings reflect antemortem anatomy rather than postmortem tissue configuration. Such studies could also incorporate direct imaging-based measurements of visceral and subcutaneous adipose compartments, allowing the relationships among BMI, waist circumference, ASFT, regional adiposity, and microscopic appendiceal adipose accumulation to be examined more directly. This integrated radiological–histopathological approach would provide a stronger basis for interpreting appendiceal structural variation than can be achieved from postmortem histology alone.

### 4.8. Conclusions

In this prospective cross-sectional forensic autopsy cohort, advancing age was associated with lower lymphoid nodule scores in the primary model, although this association no longer reached conventional statistical significance after additional adjustment for natural versus non-natural cause of death. Age-related associations with fibrosis and submucosal adipose accumulation were more consistent across sensitivity analyses. The association between age and fibrosis varied across severity thresholds, with a larger point estimate at the severe threshold. Age was also associated with greater luminal narrowing in the primary model; however, this association was less robust after additional adjustment for cause of death and in the three-level sensitivity analysis and should therefore be interpreted cautiously.

Adiposity-related associations were less uniform. BMI showed only a weak unadjusted association with submucosal adipose accumulation, and waist circumference was not significantly associated with the evaluated histopathological outcomes. In contrast, greater ASFT was associated with higher luminal-status and submucosal-adipose-accumulation scores after adjustment for age and sex. No consistent sex-related differences were identified. The SM/MP ratio showed no detectable association with age or the evaluated adiposity measures and should be regarded as an exploratory morphometric finding.

Taken together, these findings demonstrate substantial heterogeneity in appendiceal histological features across adult forensic autopsy cases. They provide descriptive morphological context for the interpretation of appendiceal tissue when it is examined during medico-legal assessment, but they do not establish diagnostic thresholds, distinguish physiological from pathological changes with known accuracy, or demonstrate that routine appendiceal histology improves cause-of-death determination. The findings should therefore be regarded as descriptive and hypothesis-generating and require confirmation in larger multicenter cohorts incorporating more detailed postmortem controls, disease comparator groups, quantitative digital morphometry, direct assessment of regional adiposity, and matched antemortem imaging.

## Figures and Tables

**Table 1:** Demographic, anthropometric, and forensic characteristics of the study population.

Parameter	Total (*n* = 101)	Male (*n* = 75)	Female (*n* = 26)
Age (years), mean ± SD	44.42 ± 18.47	45.97 ± 19.09	39.92 ± 16.04
Age range (min–max)	18–83	18–83	19–72
BMI (kg/m^2^), mean ± SD	25.92 ± 4.54	25.77 ± 4.26	26.37 ± 5.32
Waist circumference (cm), mean ± SD	89.47 ± 14.29	89.11 ± 14.23	90.50 ± 14.69
ASFT (cm), mean ± SD	3.77 ± 2.34	3.74 ± 2.41	3.83 ± 2.15
PMI (h), mean ± SD	5.07 ± 1.78	5.07 ± 1.82	5.08 ± 1.70
PMI range (h)	2–9	2–9	3–9
Cause of death, *n* (%)			
Asphyxia	33 (32.7)	20 (26.7)	13 (50.0)
Hanging	32 (31.7)	20 (26.7)	12 (46.2)
Drowning	1 (1.0)	0	1 (3.8)
Natural death	32 (31.7)	24 (32.0)	8 (30.8)
Cardiovascular disease	30 (29.7)	23 (30.7)	7 (26.9)
Cerebral hemorrhage	1 (1.0)	1 (1.3)	0
Epilepsy	1 (1.0)	0	1 (3.8)
Trauma	25 (24.8)	23 (30.7)	2 (7.7)
Firearm injury	15 (14.9)	15 (20.0)	0
Sharp-force injury	5 (5.0)	4 (5.3)	1 (3.8)
Blunt head trauma	3 (3.0)	2 (2.7)	1 (3.8)
Blunt chest trauma	2 (2.0)	2 (2.7)	0
Poisoning	10 (9.9)	7 (9.3)	3 (11.5)
Pharmaceutical drug poisoning	5 (5.0)	3 (4.0)	2 (7.7)
Carbon monoxide poisoning	4 (4.0)	3 (4.0)	1 (3.8)
Illicit drug intoxication	1 (1.0)	1 (1.3)	0
Other external cause	1 (1.0)	1 (1.3)	0
Electrocution	1 (1.0)	1 (1.3)	0

ASFT, abdominal subcutaneous fat thickness; BMI, body mass index; PMI, postmortem interval; SD, standard deviation. Percentages in the Total column were calculated using *n* = 101; percentages in the sex-specific columns were calculated using the corresponding column totals (male *n* = 75; female *n* = 26).

**Table 2:** Histopathological parameters.

Parameter	Score	Description	*n* (%)
Lymphoid nodules	0	Absent	35 (34.7)
	1	1–2 nodules	37 (36.6)
	2	3–4 nodules	21 (20.8)
	3	≥5 nodules	8 (7.9)
Fibrosis	0	Absent	2 (2.0)
	1	Mild	44 (43.6)
	2	Moderate	34 (33.7)
	3	Severe	21 (20.8)
Luminal status	0	Normal	4 (4.0)
	1	Mild narrowing	28 (27.7)
	2	Marked narrowing	52 (51.5)
	3	Severe narrowing/near-obliteration	17 (16.8)
Submucosal adipose accumulation	0	Absent/minimal	11 (10.9)
	1	Mild	22 (21.8)
	2	Moderate	43 (42.6)
	3	Severe	25 (24.8)

**Table 3:** Sex-based comparison of histopathological parameters.

Parameter	Male (*n* = 75), Median (IQR)	Female (*n* = 26), Median (IQR)	*p* Value
Lymphoid nodules	1 (0–1)	2 (0–2)	0.096
Fibrosis	2 (1–2)	2 (1–2)	0.816
Luminal status	2 (1–2)	2 (2–2)	0.432
Submucosal adipose accumulation	2 (1–3)	2 (1–2)	0.217
SM/MP ratio	1.16 (0.90–1.44)	1.14 (0.93–1.33)	0.840

Mann–Whitney U test was used for all comparisons. IQR, interquartile range; SM/MP, submucosa-to-muscularis propria ratio.

**Table 4:** Correlation of adiposity parameters with histopathological findings.

Outcome	BMI, ρ (*p*)	Waist Circumference, ρ (*p*)	ASFT, ρ (*p*)
Lymphoid nodules	−0.142 (0.156)	−0.147 (0.142)	−0.137 (0.171)
Fibrosis	−0.040 (0.688)	0.007 (0.944)	−0.065 (0.520)
Luminal status	0.165 (0.100)	0.193 (0.053)	0.218 (0.029)
Submucosal adipose accumulation	0.196 (0.049)	0.155 (0.122)	0.232 (0.020)
SM/MP ratio	0.048 (0.637)	0.052 (0.609)	0.076 (0.449)

BMI, body mass index; ASFT, abdominal subcutaneous fat thickness; SM/MP, submucosa-to-muscularis propria ratio; ρ, Spearman’s rank correlation coefficient. All analyses included 101 cases.

**Table 5:** Primary multivariable ordinal regression models for histopathological outcomes.

Outcome	Covariate/Cumulative Threshold	OR (95% CI)	*p* Value
Lymphoid nodules	Age, per year	0.971 (0.951–0.992)	0.007
	BMI, per kg/m^2^	0.941 (0.865–1.024)	0.159
	Female: score > 0 vs. 0	0.827 (0.308–2.219)	0.705
	Female: score > 1 vs. ≤1	4.401 (1.627–11.907)	0.004
	Female: score 3 vs. ≤2	1.652 (0.359–7.610)	0.519
Fibrosis	Age: moderate/severe vs. absent/mild	1.039 (1.013–1.065)	0.003
	Age: severe vs. absent/mild/moderate	1.087 (1.050–1.125)	<0.001
	Female sex	1.340 (0.546–3.284)	0.523
	BMI, per kg/m^2^	0.921 (0.841–1.009)	0.077
Luminal status	Age, per year	1.022 (1.001–1.044)	0.038
	Female sex	1.571 (0.672–3.673)	0.297
	BMI, per kg/m^2^	1.060 (0.976–1.152)	0.168
Submucosal adipose accumulation	Age, per year	1.027 (1.007–1.048)	0.010
	Female sex	0.648 (0.280–1.497)	0.309
	BMI, per kg/m^2^	1.076 (0.987–1.173)	0.095

All models included *n* = 101 cases. Male sex was the reference category. ORs > 1 indicate greater odds of membership in a higher histopathological score category. Lymphoid nodule score was analyzed using a partial proportional-odds model with threshold-specific coefficients for sex. Initial proportional-odds test: *p* = 0.006; residual test after partial proportional-odds specification: *p* = 0.205. AIC = 245.41; BIC = 266.33. Fibrosis scores 0 and 1 were combined before multivariable modeling because only two cases had a score of 0. Age was modeled with threshold-specific coefficients. Initial proportional-odds test for the three-level outcome: *p* = 0.039; residual test after partial proportional-odds specification: *p* = 0.691. AIC = 190.81; BIC = 206.50. Standard proportional-odds ordinal logistic regression was used. The proportional-odds assumption was satisfied for luminal status (*p* = 0.300; AIC = 231.43; BIC = 247.12) and submucosal adipose accumulation (*p* = 0.274; AIC = 258.60; BIC = 274.29).

**Table 6:** Age- and sex-adjusted associations of adiposity measures with histopathological outcomes.

Histopathological Outcome	BMI, OR (95% CI); *p*	Waist Circumference, OR (95% CI); *p*	ASFT, OR (95% CI); *p*
Lymphoid nodules	0.941 (0.865–1.024); 0.159	0.982 (0.957–1.008); 0.175	0.908 (0.773–1.067); 0.243
Fibrosis	0.921 (0.841–1.009); 0.077	0.988 (0.961–1.016); 0.416	0.886 (0.744–1.055); 0.173
Luminal status	1.060 (0.976–1.152); 0.168	1.025 (0.998–1.052); 0.067	1.212 (1.027–1.431); 0.023
Submucosal adipose accumulation	1.076 (0.987–1.173); 0.095	1.012 (0.985–1.040); 0.379	1.203 (1.022–1.416); 0.026

ORs represent the effect per 1 kg/m^2^ increase in BMI, 1 cm increase in waist circumference, or 1 cm increase in ASFT. Each adiposity measure was entered in a separate model adjusted for age and sex (*n* = 101). Male sex was the reference category. Lymphoid nodule models used partial proportional-odds regression with threshold-specific coefficients for sex. For fibrosis, scores 0 and 1 were combined into an absent/mild category, and age was modeled with threshold-specific coefficients. Standard proportional-odds regression was used for luminal status and submucosal adipose accumulation. BMI, body mass index; ASFT, abdominal subcutaneous fat thickness; OR, odds ratio; CI, confidence interval.

**Table 7:** PMI-adjusted sensitivity analyses of histopathological outcomes.

Histopathological Outcome	Age, OR (95% CI); *p*	PMI, OR (95% CI); *p*
Lymphoid nodules	0.970 (0.949–0.992); 0.008	1.043 (0.839–1.298); 0.702
Fibrosis	1.039 (1.012–1.066); 0.004 ^1^/1.087 (1.050–1.126); <0.001 ^2^	0.994 (0.797–1.240); 0.958
Luminal status	1.021 (0.999–1.044); 0.057	0.989 (0.789–1.238); 0.921
Submucosal adipose accumulation	1.029 (1.008–1.051); 0.007	0.921 (0.747–1.135); 0.440

Models included age, sex, BMI, and PMI (*n* = 101); only age and PMI coefficients are displayed for conciseness. Male sex was the reference category. For lymphoid nodules, a partial proportional-odds model with threshold-specific coefficients for sex was used. Fibrosis scores 0 and 1 were combined into an absent/mild category, and age was modeled with threshold-specific effects: ^1^ moderate/severe versus absent/mild; ^2^ severe versus absent/mild/moderate. For the luminal-status sensitivity analysis, the normal-open and mild-narrowing categories were combined because only four cases had a completely open lumen. The resulting three-level outcome satisfied the proportional-odds assumption (*p* = 0.302). For comparison, in the same three-level luminal model without PMI, the age estimate was OR = 1.021 (95% CI 1.000–1.042; *p* = 0.051). PMI, postmortem interval; BMI, body mass index; OR, odds ratio; CI, confidence interval.

**Table 8:** Distribution of histopathological scores according to age group.

Histopathological Outcome/Score	18–39 Years (*n* = 49), *n* (%)	40–59 Years (*n* = 29), *n* (%)	≥60 Years (*n* = 23), *n* (%)	*p* Value *
**Lymphoid nodules**				**0.012**
Score 0	14 (28.6)	10 (34.5)	11 (47.8)	
Score 1	14 (28.6)	12 (41.4)	11 (47.8)	
Score 2	13 (26.5)	7 (24.1)	1 (4.3)	
Score 3	8 (16.3)	0 (0.0)	0 (0.0)	
**Fibrosis**				**<0.001**
Score 0	2 (4.1)	0 (0.0)	0 (0.0)	
Score 1	25 (51.0)	15 (51.7)	4 (17.4)	
Score 2	20 (40.8)	9 (31.0)	5 (21.7)	
Score 3	2 (4.1)	5 (17.2)	14 (60.9)	
**Luminal status**				**0.312**
Score 0	3 (6.1)	1 (3.4)	0 (0.0)	
Score 1	17 (34.7)	6 (20.7)	5 (21.7)	
Score 2	21 (42.9)	16 (55.2)	15 (65.2)	
Score 3	8 (16.3)	6 (20.7)	3 (13.0)	
**Submucosal adipose accumulation**				**0.007**
Score 0	7 (14.3)	4 (13.8)	0 (0.0)	
Score 1	13 (26.5)	8 (27.6)	1 (4.3)	
Score 2	20 (40.8)	10 (34.5)	13 (56.5)	
Score 3	9 (18.4)	7 (24.1)	9 (39.1)	

* *p* values were obtained using the Kruskal–Wallis test across the three age groups. Age-group analyses were exploratory and descriptive; age was retained as a continuous variable in the primary regression analyses.

## Data Availability

The data are not publicly available due to confidentiality and institutional regulations governing forensic autopsy data. Data supporting the findings of this study are available from the corresponding author upon reasonable request and subject to institutional approval.

## Supplementary Materials

The following supporting information can be downloaded at: https://www.mdpi.com/article/10.3390/diagnostics16213495/s1, Figure S1: Flow diagram of case selection and analytical inclusion; Table S1: Complete age- and sex-adjusted ordinal regression models for BMI, waist circumference, and ASFT; Table S2: Complete PMI-adjusted sensitivity models; Table S3: Sensitivity analyses additionally adjusted for natural versus non-natural cause of death.
