# Supplementary material for: Histopathological Spectrum of the Human Appendix with Aging and Adiposity: A Forensic Autopsy Study
Source: Diagnostics (Basel). 2026 Oct 25;16(21):3495. doi: 10.3390/diagnostics16213495 (PMC13762253; doi:10.3390/diagnostics16213495)
Supplement: Supplementary file 1 [file diagnostics-16-03495-s001.zip › STROBE Appendix Autopsy.pdf]

STROBE Statement—Checklist of items that should be included in reports of ***cross-sectional studies***

Manuscript: Histopathological Spectrum of the Human Appendix with Aging and Adiposity: A Forensic Autopsy Study

	Item No	Recommendation	Reported on page/section
**Title and abstract**	1	(*a*) Indicate the study's design with a commonly used term in the title or the abstract	Abstract (Methods): study identified as a prospective cross-sectional observational autopsy study.
		(*b*) Provide in the abstract an informative and balanced summary of what was done and what was found	Abstract (Background/Methods/Results/Conclusions).
		**Introduction**	
Background/rationale	2	Explain the scientific background and rationale for the investigation being reported	Section 1, Introduction, paragraphs 1–4.
Objectives	3	State specific objectives, including any prespecified hypotheses	Section 1, Introduction, final paragraph (study objective).
		**Methods**	
Study design	4	Present key elements of study design early in the paper	Section 2.1, Study Design and Study Population.
Setting	5	Describe the setting, locations, and relevant dates, including periods of recruitment, exposure, follow-up, and data collection	Section 2.1: Council of Forensic Medicine, Konya Group Chairmanship; 01 January–31 March 2026.
Participants	6	(*a*) Give the eligibility criteria, and the sources and methods of selection of participants	Section 2.1: eligibility/exclusion criteria and consecutive selection of adult forensic autopsy cases.
Variables	7	Clearly define all outcomes, exposures, predictors, potential confounders, and effect modifiers. Give diagnostic criteria, if applicable	Sections 2.2–2.6: demographic/anthropometric variables, PMI, cause of death, histopathological outcomes, SM/MP ratio, and covariates are defined.
Data sources/ measurement	8*	For each variable of interest, give sources of data and details of methods of assessment (measurement). Describe comparability of assessment methods if there is more than one group	Sections 2.2–2.5: anthropometry, tissue sampling, histopathology, ordinal scoring, and SM/MP

	Item No	Recommendation	Reported on page/section
			morphometry.
Bias	9	Describe any efforts to address potential sources of bias	Sections 2.1–2.6 and 4.6: consecutive sampling, standardized protocol, blinded histological scoring, consensus review, artifact exclusion, PMI and cause-of-death sensitivity analyses, and explicit discussion of residual confounding and other limitations.
Study size	10	Explain how the study size was arrived at	Section 2.1: study size determined by consecutive eligible cases available during the predefined study period; no formal a priori sample-size calculation.
Quantitative variables	11	Explain how quantitative variables were handled in the analyses. If applicable, describe which groupings were chosen and why	Section 2.6: principal quantitative covariates were retained as continuous. Predefined ordinal histopathological scores were used; fibrosis and luminal categories were collapsed only for specified regression/sensitivity analyses. Exploratory age strata (18–39, 40–59, and ≥60 years) and the rationale for these cut points are described.
Statistical methods	12	(*a*) Describe all statistical methods, including those used to control for confounding	Section 2.6, Statistical Analysis; Tables 4–7 and Supplementary Tables S1–S3: correlation analyses, proportional- and partial proportional-odds models, covariate adjustment,

	Item No	Recommendation	Reported on page/section
			separate adiposity models, model diagnostics, and sensitivity analyses.
		(*b*) Describe any methods used to examine subgroups and interactions	Sections 2.6, 3.3, and 3.7: sex-based comparisons and exploratory age-group analyses were performed. No formal interaction analyses were performed.
		(*c*) Explain how missing data were addressed	Sections 2.1 and 2.6: no missing data; all 101 analysed cases contributed to the reported analyses.
		(*d*) If applicable, describe analytical methods taking account of sampling strategy	Not applicable: consecutive eligible-case sampling was used; no complex survey or probability-sampling design.
		(*e*) Describe any sensitivity analyses	Sections 2.6 and 3.6: sensitivity analyses included PMI-adjusted models and additional adjustment for natural versus non-natural cause of death; the three-level luminal-status sensitivity model is also described. Table 7 and Supplementary Tables S2–S3.
		**Results**	
Participants	13*	(a) Report numbers of individuals at each stage of study—eg numbers potentially eligible, examined for eligibility, confirmed eligible, included in the study, completing follow-up, and analysed	Section 2.1 and Results Section 3.1: 136 adult forensic autopsy cases were assessed for eligibility; 35 were excluded; 101 eligible cases were included in the

	Item No	Recommendation	Reported on page/section
			final analytical cohort, and all 101 were analysed.
		(b) Give reasons for non-participation at each stage	Section 2.1: 35 cases were excluded: abdominal trauma (n = 8), penetrating or sharp-force abdominal injury (n = 6), advanced appendiceal putrefaction/autolysis precluding reliable histopathological evaluation (n = 4), history of colorectal malignancy (n = 3), and documented diabetes mellitus (n = 14).
		(c) Consider use of a flow diagram	Supplementary Figure S1: study flow diagram showing 136 cases assessed for eligibility, 35 excluded with reasons, 101 included in the final analytical cohort, and 101 analysed.
Descriptive data	14*	(a) Give characteristics of study participants (eg demographic, clinical, social) and information on exposures and potential confounders	Section 3.1 and Table 1: demographic, anthropometric, PMI, and cause-of-death characteristics.
		(b) Indicate number of participants with missing data for each variable of interest	Sections 2.1 and 2.6: no missing data for analysed variables (n = 101 complete cases).
Outcome data	15*	Report numbers of outcome events or summary measures	Sections 3.2–3.5 and 3.7;

	Item No	Recommendation	Reported on page/section
			Tables 2–4 and 8: distributions and summary measures of histopathological outcomes and SM/MP ratio, including exploratory age-stratified histopathological distributions.
Main results	16	(*a*) Give unadjusted estimates and, if applicable, confounder-adjusted estimates and their precision (eg, 95% confidence interval). Make clear which confounders were adjusted for and why they were included	Section 3.6; Tables 5–7; Supplementary Tables S1–S3: unadjusted and adjusted estimates, ORs, 95% CIs, exact p values, covariates, reference categories, model diagnostics, and PMI/cause-of-death sensitivity analyses.
		(*b*) Report category boundaries when continuous variables were categorized	Principal continuous covariates were retained as continuous in the primary analyses. Ordinal outcome boundaries are defined in Section 2.4, and category collapsing for specific models is described in Section 2.6. For exploratory analyses, age was categorized as 18–39, 40–59, and ≥60 years; the cut-point rationale is provided in Section 2.6.
		(*c*) If relevant, consider translating estimates of relative risk into absolute risk for a meaningful time period	Not applicable: no relative-risk estimates requiring translation into absolute risk were reported.
Other analyses	17	Report other analyses done—eg analyses of subgroups and interactions, and sensitivity analyses	Sections 3.3, 3.6, and 3.7; Tables 3, 6–8; Supplementary Tables S1–S3: sex comparisons, separate adiposity models,

	Item No	Recommendation	Reported on page/section
			collinearity assessment, PMI sensitivity analyses, cause-of-death sensitivity analyses, and exploratory age-group analyses.
		**Discussion**	
Key results	18	Summarise key results with reference to study objectives	Section 4, Discussion opening paragraph; Sections 4.1–4.5; Section 4.8, Conclusion.
Limitations	19	Discuss limitations of the study, taking into account sources of potential bias or imprecision. Discuss both direction and magnitude of any potential bias	Section 4.6, Strengths and Limitations.
Interpretation	20	Give a cautious overall interpretation of results considering objectives, limitations, multiplicity of analyses, results from similar studies, and other relevant evidence	Sections 4.1–4.8: cautious interpretation of cross-sectional associations, adiposity findings, PMI and cause-of-death sensitivity analyses, multiplicity, residual confounding, and the absence of diagnostic comparator groups or validated diagnostic thresholds.
Generalisability	21	Discuss the generalisability (external validity) of the study results	Section 4.6 (single-center cohort and selection limitations) and Section 4.7 (future multicenter, sex-balanced validation).
		**Other information**	
Funding	22	Give the source of funding and the role of the funders for the present study and, if applicable, for the original study on which the present article is based	Funding statement: no specific grant from public, commercial, or not-for-profit funding agencies.

*Give information separately for exposed and unexposed groups.

Note: An Explanation and Elaboration article discusses each checklist item and gives methodological background and published examples of transparent reporting. The STROBE checklist is best used in conjunction with this article (freely available on the Web sites of PLoS Medicine at http://www.plosmedicine.org/, Annals of Internal Medicine at http://www.annals.org/, and Epidemiology at http://www.epidem.com/). Information on the STROBE Initiative is available at www.strobe-statement.org.

Note: Section references are provided because final manuscript pagination may change during journal formatting.
