# Supplementary material for: Histopathological Spectrum of the Human Appendix with Aging and Adiposity: A Forensic Autopsy Study
Source: Diagnostics (Basel). 2026 Oct 25;16(21):3495. doi: 10.3390/diagnostics16213495 (PMC13762253; doi:10.3390/diagnostics16213495)
Supplement: Supplementary file 1 [file diagnostics-16-03495-s001.zip › Supplementary Table S1.pdf]

Supplementary Table S1. Complete age- and sex-adjusted ordinal regression models for BMI, waist circumference, and ASFT

Outcome / model	Covariate	OR (95% CI)	p
**Lymphoid – BMI**	Age, per year	0.971 (0.951–0.992)	0.007
	BMI, per kg/m²	0.941 (0.865–1.024)	0.159
	Female: score >0 vs 0	0.827 (0.308–2.219)	0.705
	Female: score >1 vs ≤1	4.401 (1.627–11.907)	0.004
	Female: score 3 vs ≤2	1.652 (0.359–7.610)	0.519
**Lymphoid – Waist**	Age, per year	0.971 (0.951–0.992)	0.007
	Waist, per cm	0.982 (0.957–1.008)	0.175
	Female: score >0 vs 0	0.808 (0.302–2.160)	0.671
	Female: score >1 vs ≤1	4.317 (1.603–11.631)	0.004
	Female: score 3 vs ≤2	1.658 (0.360–7.627)	0.516
**Lymphoid – ASFT**	Age, per year	0.970 (0.950–0.991)	0.005
	ASFT, per cm	0.908 (0.773–1.067)	0.243
	Female: score >0 vs 0	0.794 (0.298–2.116)	0.644
	Female: score >1 vs ≤1	4.242 (1.582–11.372)	0.004
	Female: score 3 vs ≤2	1.619 (0.353–7.432)	0.535
**Fibrosis – BMI**	Age: moderate/severe vs absent/mild	1.039 (1.013–1.065)	0.003
	Age: severe vs lower categories	1.087 (1.050–1.125)	0.000002
	Female sex	1.340 (0.546–3.284)	0.523
	BMI, per kg/m²	0.921 (0.841–1.009)	0.077
**Fibrosis – Waist**	Age: moderate/severe vs absent/mild	1.035 (1.010–1.061)	0.006
	Age: severe vs lower categories	1.085 (1.049–1.122)	0.000003
	Female sex	1.304 (0.537–3.169)	0.557
	Waist, per cm	0.988 (0.961–1.016)	0.416
**Fibrosis – ASFT**	Age: moderate/severe vs absent/mild	1.035 (1.010–1.061)	0.005
	Age: severe vs lower categories	1.085 (1.049–1.123)	0.000003
	Female sex	1.291 (0.529–3.151)	0.574
	ASFT, per cm	0.886 (0.744–1.055)	0.173
**Luminal status – BMI**	Age, per year	1.022 (1.001–1.044)	0.038
	Female sex	1.571 (0.672–3.673)	0.297
	BMI, per kg/m²	1.060 (0.976–1.152)	0.168
**Luminal status – Waist**	Age, per year	1.022 (1.001–1.043)	0.042
	Female sex	1.544 (0.660–3.611)	0.316
	Waist, per cm	1.025 (0.998–1.052)	0.067
**Luminal status – ASFT**	Age, per year	1.024 (1.003–1.045)	0.025
	Female sex	1.627 (0.694–3.814)	0.263
	ASFT, per cm	1.212 (1.027–1.431)	0.023
**Submucosal adipose – BMI**	Age, per year	1.027 (1.007–1.048)	0.010
	Female sex	0.648 (0.280–1.497)	0.309
	BMI, per kg/m²	1.076 (0.987–1.173)	0.095
**Submucosal adipose – Waist**	Age, per year	1.028 (1.007–1.049)	0.007
	Female sex	0.691 (0.303–1.580)	0.381
	Waist, per cm	1.012 (0.985–1.040)	0.379
**Submucosal adipose – ASFT**	Age, per year	1.029 (1.008–1.050)	0.006
	Female sex	0.677 (0.294–1.559)	0.359

Outcome / model	Covariate	OR (95% CI)	p
	ASFT, per cm	1.203 (1.022–1.416)	0.026

Model fit: Lymphoid BMI AIC/BIC = **245.41/266.33**; waist = **245.57/266.49**; ASFT = **246.06/266.98**. Fibrosis BMI = **190.81/206.50**; waist = **193.35/209.05**; ASFT = **192.13/207.82**. Luminal BMI = **231.43/247.12**; waist = **229.94/245.63**; ASFT = **228.07/243.76**. Submucosal adipose BMI = **258.60/274.29**; waist = **260.67/276.36**; ASFT = **256.42/272.11**.

Footnote: All models included n=101 with no missing data. Male sex was the reference category. Lymphoid models used partial proportional odds with threshold-specific sex coefficients; fibrosis scores 0 and 1 were combined and age had threshold-specific effects. Luminal-status and submucosal-adipose models used standard proportional odds. The primary proportional-odds diagnostics were: lymphoid initial p=0.006, residual after partial specification p=0.205; fibrosis initial p=0.039, residual p=0.691; luminal status p=0.300; submucosal adipose accumulation p=0.274. The same outcome-specific model structure was retained for the alternate adiposity models.
