# Supplementary material for: Histopathological Spectrum of the Human Appendix with Aging and Adiposity: A Forensic Autopsy Study
Source: Diagnostics (Basel). 2026 Oct 25;16(21):3495. doi: 10.3390/diagnostics16213495 (PMC13762253; doi:10.3390/diagnostics16213495)
Supplement: Supplementary file 1 [file diagnostics-16-03495-s001.zip › Supplementary Table S2.pdf]

Supplementary Table S2. Complete PMI-adjusted sensitivity models

Outcome	Covariate	OR (95% CI)	p
**Lymphoid nodules**	Age, per year	0.970 (0.949–0.992)	0.008
	BMI, per kg/m²	0.940 (0.864–1.023)	0.154
	PMI, per hour	1.043 (0.839–1.298)	0.702
	Female: score >0 vs 0	0.813 (0.301–2.193)	0.683
	Female: score >1 vs ≤1	4.363 (1.609–11.831)	0.004
	Female: score 3 vs ≤2	1.656 (0.359–7.639)	0.518
**Fibrosis**	Age: moderate/severe vs absent/mild	1.039 (1.012–1.066)	0.004
	Age: severe vs lower categories	1.087 (1.050–1.126)	0.000003
	Female sex	1.343 (0.545–3.308)	0.521
	BMI, per kg/m²	0.921 (0.841–1.009)	0.077
	PMI, per hour	0.994 (0.797–1.240)	0.958
**Luminal status**	Age, per year	1.021 (0.999–1.044)	0.057
	Female sex	1.486 (0.631–3.499)	0.365
	BMI, per kg/m²	1.056 (0.971–1.149)	0.203
	PMI, per hour	0.989 (0.789–1.238)	0.921
**Submucosal adipose**	Age, per year	1.029 (1.008–1.051)	0.007
	Female sex	0.641 (0.277–1.487)	0.300
	BMI, per kg/m²	1.079 (0.989–1.176)	0.087
	PMI, per hour	0.921 (0.747–1.135)	0.440

Model fit: lymphoid AIC/BIC = **247.26/270.80**; fibrosis = **192.81/211.12**; luminal status = **208.27/223.96**; submucosal adipose accumulation = **260.00/278.31**.

For the luminal PMI sensitivity model, scores 0 and 1 were combined because only four cases had a completely open lumen; the resulting three-level model satisfied the proportional-odds assumption (**p=0.302**). All sensitivity models included age, sex, BMI, and PMI, n=101, with no missing data.
