# Supplementary material for: Histopathological Spectrum of the Human Appendix with Aging and Adiposity: A Forensic Autopsy Study
Source: Diagnostics (Basel). 2026 Oct 25;16(21):3495. doi: 10.3390/diagnostics16213495 (PMC13762253; doi:10.3390/diagnostics16213495)
Supplement: Supplementary file 1 [file diagnostics-16-03495-s001.zip › Supplementary Table S3.pdf]

Supplementary Table S3. Sensitivity analyses additionally adjusted for natural versus non-natural cause of death

**Outcome**	**Covariate**	**OR (95% CI)**	**p**
Lymphoid nodules	Age, per year	0.978 (0.955–1.001)	0.063
	BMI, per kg/m²	0.944 (0.868–1.027)	0.182
	Natural death vs non-natural	0.542 (0.222–1.323)	0.179
	Female: score >0 vs 0	0.841 (0.311–2.270)	0.732
	Female: score >1 vs ≤1	4.649 (1.708–12.657)	0.003
	Female: score 3 vs ≤2	1.767 (0.382–8.168)	0.466
Fibrosis	Age: moderate/severe vs absent/mild	1.037 (1.009–1.065)	0.009
	Age: severe vs lower categories	1.085 (1.046–1.125)	<0.001
	Female sex	1.324 (0.539–3.255)	0.540
	BMI, per kg/m²	0.920 (0.839–1.008)	0.073
	Natural death vs non-natural	1.160 (0.458–2.940)	0.754
Luminal status	Age, per year	1.020 (0.996–1.044)	0.109
	Female sex	1.559 (0.666–3.647)	0.306
	BMI, per kg/m²	1.060 (0.975–1.152)	0.173
	Natural death vs non-natural	1.228 (0.488–3.091)	0.663
Submucosal adipose accumulation	Age, per year	1.028 (1.006–1.052)	0.015
	Female sex	0.649 (0.280–1.502)	0.313
	BMI, per kg/m²	1.076 (0.988–1.173)	0.094
	Natural death vs non-natural	0.905 (0.385–2.125)	0.818

All models included n = 101 cases with no missing data. Male sex and non-natural death were the reference categories. Cause of death was dichotomized as natural versus non-natural death and entered together with age, sex, and BMI. Lymphoid nodule score was analyzed using a partial proportional-odds model with threshold-specific coefficients for sex. For fibrosis, scores 0 and 1 were combined into an absent/mild category, and age was modeled with threshold-specific effects. Standard proportional-odds regression was used for luminal status and submucosal adipose accumulation. The same outcome-specific model specification as in the primary analyses was retained.

Model fit: lymphoid nodules AIC/BIC = 245.59/269.13; fibrosis = 192.71/211.02; luminal status = 233.24/251.55; submucosal adipose accumulation = 260.55/278.85. BMI, body mass index; OR, odds ratio; CI, confidence interval.
